# The Visceral Adiposity Index in Non-Alcoholic Fatty Liver Disease and Liver Fibrosis—Systematic Review and Meta-Analysis

**DOI:** 10.3390/biomedicines9121890

**Published:** 2021-12-13

**Authors:** Abdulrahman Ismaiel, Ayman Jaaouani, Daniel-Corneliu Leucuta, Stefan-Lucian Popa, Dan L. Dumitrascu

**Affiliations:** 1 2nd Department of Internal Medicine, “Iuliu Hatieganu” University of Medicine and Pharmacy, 400006 Cluj-Napoca, Romania; abdulrahman.ismaiel@yahoo.com (A.I.); popa.stefan@umfcluj.ro (S.-L.P.); ddumitrascu@umfcluj.ro (D.L.D.); 2 Faculty of Medicine, “Iuliu Hatieganu” University of Medicine and Pharmacy, 400006 Cluj-Napoca, Romania; ayman.jaaouani.aj@gmail.com; 3 Department of Medical Informatics and Biostatistics, “Iuliu Hatieganu” University of Medicine and Pharmacy, 400349 Cluj-Napoca, Romania

**Keywords:** non-alcoholic fatty liver disease (NAFLD), non-alcoholic steatohepatitis (NASH), hepatic steatosis, liver fibrosis, visceral adiposity index (VAI), liver biopsy, noninvasive markers

## Abstract

(1) Background: In order to avoid a liver biopsy in non-alcoholic fatty liver disease (NAFLD), several noninvasive biomarkers have been studied lately. Therefore, we aimed to evaluate the visceral adiposity index (VAI) in NAFLD and liver fibrosis, in addition to its accuracy in predicting NAFLD and NASH. (2) Methods: We searched PubMed, Embase, Scopus, and Cochrane Library, identifying observational studies assessing the VAI in NAFLD and liver fibrosis. QUADAS-2 was used to evaluate the quality of included studies. The principal summary outcomes were mean difference (MD) and area under the curve (AUC). (3) Results: A total of 24 studies were included in our review. VAI levels were significantly increased in NAFLD (biopsy-proven and ultrasound-diagnosed), simple steatosis vs. controls, and severe steatosis vs. simple steatosis. However, no significant MD was found according to sex, liver fibrosis severity, simple vs. moderate and moderate vs. severe steatosis, pediatric NAFLD, and NASH patients. The VAI predicted NAFLD (AUC 0.767) and NASH (AUC 0.732). (4) Conclusions: The VAI has a predictive value in diagnosing NAFLD and NASH, with significantly increased values in adult NAFLD patients, simple steatosis compared to controls, and severe steatosis compared to simple steatosis.

## 1. Introduction

Nonalcoholic fatty liver disease (NAFLD) is a multi-system disease, being mainly a liver pathology involving excessive hepatic fat accumulation unrelated to alcohol consumption or other secondary causes of hepatic steatosis [1]. It is an emerging cause of concern and increasing clinical burden, imposing a public health challenge. NAFLD is the most common chronic liver disease and is predicted to be the most common indication for a liver transplant in Western countries by 2030, owing to a prevalence of 25% worldwide [2,3]. As of today, no therapies are approved for the management of NAFLD.

NAFLD includes a spectrum ranging from non-alcoholic fatty liver (NAFL), which can progress to non-alcoholic steatohepatitis (NASH), being associated with lower survival rates as demonstrated in long-term longitudinal studies and likely to progress, if left without intervention, to cirrhosis, liver failure, and hepatocellular carcinoma (HCC) [4,5,6]. Moreover, NAFLD is also associated with extra-hepatic complications, including a significant increase in overall mortality from cardiovascular causes, as well as an increased incidence of type 2 diabetes mellitus (T2DM) and chronic kidney disease (CKD) [7,8].

Obesity, a modifiable risk factor also common among NAFLD patients, is highly associated with lipodystrophy, adipose tissue dysfunction, metabolic syndrome (MetS), T2DM, and hepatic steatosis. Moreover, MetS is more prevalent among NAFLD patients than those without it [9]. NAFLD is more often being recognized as the hepatic manifestation of MetS, involving an interplay of adipokines released from excess visceral adipose tissue (VAT), cytokines, and inflammatory factors secreted from the macrophages residing in VAT, resulting in a chronic state of inflammation and decreased hepatic insulin extraction, all leading to insulin resistance [10,11,12,13,14].

Currently, histopathological sampling is the gold standard for differentiating NAFL from NASH as well as for liver fibrosis staging [15]. However, the procedure is invasive, with exposed sampling errors and inter-observer variability [16]. Hence, it is a critical matter to identify NAFLD using rapid, cheap, noninvasive methods with low risks for such a prevalent condition in order to evaluate the risk and prevent disease progression, and thereby complications. The current guidelines agree that risk stratification can be performed by noninvasive methods. However, no acceptable noninvasive techniques were found to differentiate between bland steatosis and steatohepatitis.

In this context, the accuracy of several anthropometric indicators, biomarkers, and complex models have been evaluated in predicting NAFLD and quantifying liver fibrosis [17]. The current guidelines recommend several noninvasive biomarkers and scores for predicting hepatic steatosis and steatohepatitis, such as the fatty liver index (FLI) and the NAFLD liver fat score [18,19]. Moreover, increased cytokeratin-18 was found to have a good predictive value for NASH from normal livers; however, it was not able to differentiate NASH from simple steatosis. Noninvasive scores of advanced fibrosis include the NAFLD fibrosis score (NFS), the fibrosis-4 index (FIB-4), the AST/ALT ratio index, and serum biomarkers such as the ELF panel, FibroMeter, FibroTest, and HepaScore [20].

Nevertheless, the current NAFLD diagnosis guidelines lack any recommendations regarding the visceral adiposity index (VAI), a scoring system based on body mass index, triglycerides, high-density lipoproteins (HDLs), and waist circumferences (WCs), probably due to inconclusive and insufficient data [21]. The VAI indirectly reflects the visceral adiposity function and is also related to insulin resistance [22]. The VAI has been recently studied as a screening tool for MetS and high-risk ‘patients’ detection [23]. It has been shown to be performant in accurately screening for MetS, making the VAI more relevant to be studied in NAFLD.

Therefore, we conducted a systematic review and meta-analysis aiming to assess the VAI in NAFLD, including hepatic steatosis and NASH, as well as quantifying liver fibrosis. We also evaluated whether the VAI can differentiate between different hepatic steatosis grades and simple steatosis from NASH.

## 2. Materials and Methods

This systematic review and meta-analysis were written as per the Preferred Reporting Items for Systematic Reviews and Meta-Analyses (PRISMA) 2020 statement [24]. The study was registered in INPLASY (International Platform of Registered Systematic Review and Meta-analysis Protocols); registration number (INPLASY2021120056) [25].

### 2.1. Data Sources and Search Strategy

We conducted a computerized search in PubMed, Embase, Scopus, and Cochrane Library electronic databases in order to identify observational studies assessing the VAI in NAFLD and liver fibrosis. The used search string is described in Supplementary Materials. Moreover, we performed a manual search for relevant missed publications through screening the references of included articles. The literature search was conducted from inception till the 19 October 2021 by two investigators (A.I. and D.C.L.) independently. In the case of discrepancies, a consensus was reached through discussion. We did not apply any filter or restrictions to duration, country, or language during the search. The titles and abstracts were then screened for eligibility, followed by full-text assessment of articles fulfilling our inclusion and exclusion criteria. Data extraction was performed by one investigator (A.J.) and verified by another (S.L.P.), while any discrepancies were resolved by confronting the source article. The extracted data included author names, publication year, country, design of the study, studied population, total sample size, NAFLD percentage, mean age, sex distribution, body mass index (BMI), NAFLD diagnosis technique, mean ± SD or median (interquartile range), area under receiver operating characteristic (AUROC) curve, and main study outcome, which were collated and presented in the manuscript text.

### 2.2. Eligibility Criteria

The inclusion criteria of original articles in our systematic review and meta-analysis were as follows: (1) observational cohort, cross-sectional, or case–control studies assessing the VAI in NAFLD and liver fibrosis; (2) hepatic steatosis confirmed histologically through a liver biopsy, or evaluated by imagistic techniques such as ultrasonography, computed tomography (CT), and magnetic resonance imaging (MRI), or by noninvasive biomarkers and scores; (3) liver fibrosis confirmed histologically, or assessed by transient elastography (FibroScan), or noninvasive biomarkers and scores; (4) human studies with no restrictions to gender, race, or ethnicity; and (5) studies published in English, German, French, or Romanian.

The exclusion criteria were as follows: (1) the presence of secondary causes of hepatic steatosis, significant alcohol consumption based on each study definition or any other cause of chronic liver disease (CLD); (2) liver cirrhosis of any etiology or end-stage liver disease patients who underwent or were awaiting liver transplantation; (3) hepatitis virus of any etiology; (4) HIV infection or use of antiretroviral therapy; (5) polycystic ovarian syndrome; and (6) editorials, letters, short surveys, commentaries, case reports, conference abstracts, review articles, practice guidelines, and abstracts published without a full article.

### 2.3. Risk of Bias Assessment in Individual Studies

Quality assessment of all included studies, evaluating the risk of bias and internal validity, was performed in a similar manner using the Quality Assessment of Diagnostic Accuracy Studies 2 (QUADAS-2) tool by two investigators (A.I. and D.C.L.) independently [26]. A consensus was reached through discussion in the presence of disagreement between the evaluations of the two investigators. The items assessed in the quality assessment tool were answered by either “yes”, “no”, or “unclear”. The eligibility of the studies was not affected by the methodological quality assessment results.

### 2.4. Summary Measures and Synthesis of Results

The data analyses of the systematic review and meta-analysis were performed using R with the Metafor package (OpenMeta [Analyst]) [27,28]. The principal summary outcomes of the VAI in NAFLD and liver fibrosis were the mean difference (MD) of the VAI and the area under the curve (AUC) evaluating the accuracy of the VAI. Between-study heterogeneity was evaluated by a χ^2^-based Q-test and I^2^. As per the recommendations of the Cochrane Handbook for identifying and measuring heterogeneity, we estimated I^2^ values of 0 to 40% as not important; 30 to 60% as moderate heterogeneity; 50 to 90% as substantial heterogeneity; and 75 to 100% as considerable heterogeneity. In studies reporting medians and interquartile ranges (IQRs), we calculated the mean and standard deviation (SD) based on them. The standard error of the AUC was calculated from the confidence interval (CI) and the point estimate. For the one study where the authors did not report the confidence intervals for the ROC curve, we proceeded to extract the data of the ROC curves using WebPlotDigitizer, version 4.5 (c 2010-2021 Ankit Rohatgi https://apps.automeris.io/wpd/ accessed on 1 November 2021), and we then computed the standard error of the mean with pROC package version 1.17.0.1 [29] using the method of Obuchowski [30]. We combined the statistics (means and standard deviations) of the groups in studies with several subgroups of NAFLD patients or control subjects, to get the statistic for the entire set of subjects (when this was missing), according to the Cochrane Handbook recommendations. Subgroup analysis was conducted according to the diagnosis method of NAFLD, adults, pediatrics, the severity of hepatic steatosis, NASH, liver fibrosis grading, diabetic/prediabetic NAFLD patients, and sex, depending on the available values from the extracted data from included studies. For all meta-analyses, we used restricted maximum likelihood random-effects models. We reported the data from each study as the estimated MD with 95% CI, lower bound, upper bound, standard error, and *p*-value, or the estimated AUC with 95% CI, lower bound, upper bound, standard error, and *p*-value. Statistical significance was considered to be achieved if the *p*-value was <0.05. The analyses were conducted if at least two studies reported the same outcome with available mean and SD, median (IQR), or AUC with lower and upper CI levels of the VAI.

### 2.5. Publication Bias, Sensitivity Analyses, and Meta-Regression

For meta-analyses that included more than 10 studies, we checked for publication bias with funnel plots and the Egger test; in the case of high heterogeneity, we did a sensitivity analysis and meta-regression analyses to explore the heterogeneity. The sensitivity analysis assessed the leave-one-out effect on heterogeneity and effect estimate, excluding high-leverage studies or outliers, which were identified with dmetar package version [31].

A random-effects metaregression was performed, using the Knapp–Hartung method to compute confidence intervals and *p*-values. Several models were built that accounted for the year of publication and for the most important QUADAS-2 bias domains identified in the risk of bias assessment of the studies. We used one variable per model to prevent overfitting since the two metaregressions had 12 and 14 included (number of studies divided by ten to find how many variables to use in the model), and only one multivariate model that included two variables, thus with some degree of overfitting. For the multivariate model, we checked for the presence of multicollinearity with variance inflation factors. The sensitivity analyses and metaregressions were performed in R environment for statistical computing and graphics, version 4.0.2 (R Foundation for Statistical Computing, Vienna, Austria), using the meta R package [32,33].

## 3. Results

### 3.1. General Results

**Figure 1:**
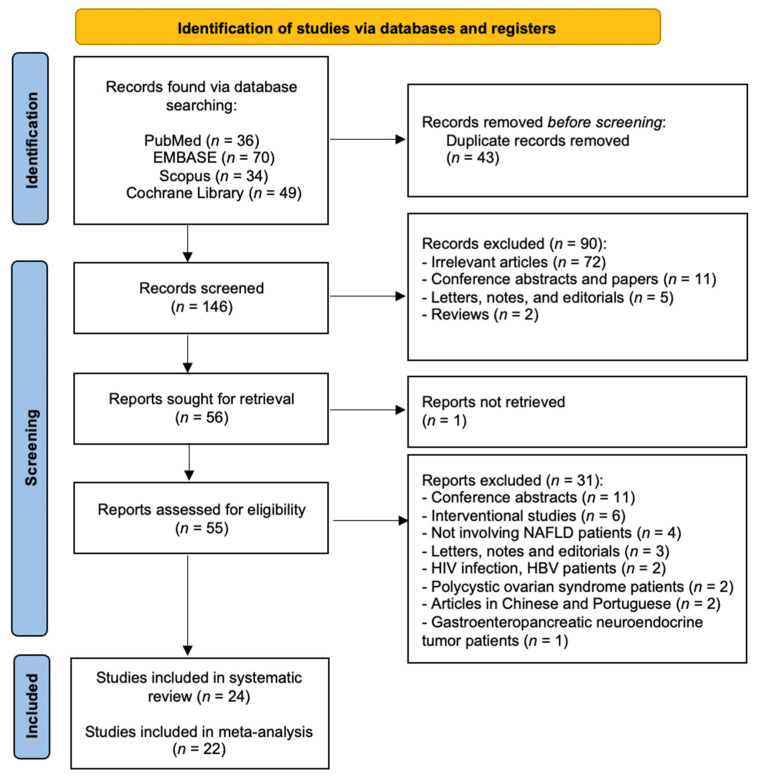
PRISMA flow diagram—identification, screening, and inclusion phases of our systematic review and meta-analysis.

The initial search yielded one hundred and eighty-nine articles (PubMed = 36 articles, EMBASE = 70 articles, Scopus = 34 articles, and Cochrane Library = 49 articles), as shown in Figure 1. A total of forty-three studies were detected as duplicates and removed. After the removal of duplicates, one hundred and forty-six articles were evaluated for inclusion and exclusion criteria fulfillment by assessing the titles and abstracts. After the first screening was performed, we excluded a total of 90 articles as follows: (1) two reviews, (2) eleven conference abstracts and papers, (3) five letters, editorials, and notes, and (4) seventy-two irrelevant studies to this review topic. We were not able to retrieve one article. Subsequently, we performed a thorough reading and evaluation of the full texts for further eligibility assessment for the remaining fifty-five articles. Of these articles, thirty-one were excluded with reasons as follows: (1) eleven articles were conference abstracts [34,35,36,37,38,39,40,41,42,43,44], (2) three articles were letters, editorials, and notes [45,46,47], (3) four did not involve NAFLD patients [48,49,50,51], (4) two studies involved HIV patients [52,53], (6) one study involved patients with gastroenteropancreatic neuroendocrine tumors [54], (7) two studies involved patients with polycystic ovarian syndrome [51,55], (8) six were interventional studies [56,57,58,59,60,61], and (9) two articles were published in Chinese and Portuguese [62,63]. The total number of articles included in the qualitative synthesis was twenty-four studies, out of which twenty-two studies were included in the quantitative synthesis [36,42,44,55,64,65,66,67,68,69,70,71,72,73,74,75,76,77,78,79,80,81,82,83].

### 3.2. Study Characteristics

A summary of the main characteristics of the included studies is presented in Supplementary Table S1. This systematic review and meta-analysis included a total number of 70,519 individuals. The sex distribution was higher for males (females—28,248 (40.1%), males—42,271 (59.9%)). NAFLD was present in 25,429 subjects (36%) of the total study sample. Nine studies were conducted in Europe (Italy *n* = 3, Spain *n* = 1, Turkey *n* = 2, Serbia *n* = 1, France *n* = 1, and Bulgaria *n* = 1), three in the Middle East (Iran *n* = 2, Egypt *n* = 1), three in Asia (China *n* = 7, Japan *n* = 1), and two in both North America (USA *n* = 1, Mexico *n* = 1) and Australia (*n* = 2).

### 3.3. Definition of NAFLD

Hepatic steatosis was assessed using ultrasonography for diagnosing NAFLD in most studies (*n* = 15) [36,55,64,67,68,70,73,75,76,77,79,80,81,82,83], while five studies used a biopsy [42,44,65,66,78]. Moreover, two studies used both [69,72]. Finally, one study used abdominal CT according to the liver/spleen attenuation ratio [71], and one study used proton magnetic resonance spectroscopy (H-MRS) in combination with steatosis biomarkers [74].

### 3.4. The VAI and NAFLD

**Figure 2:**
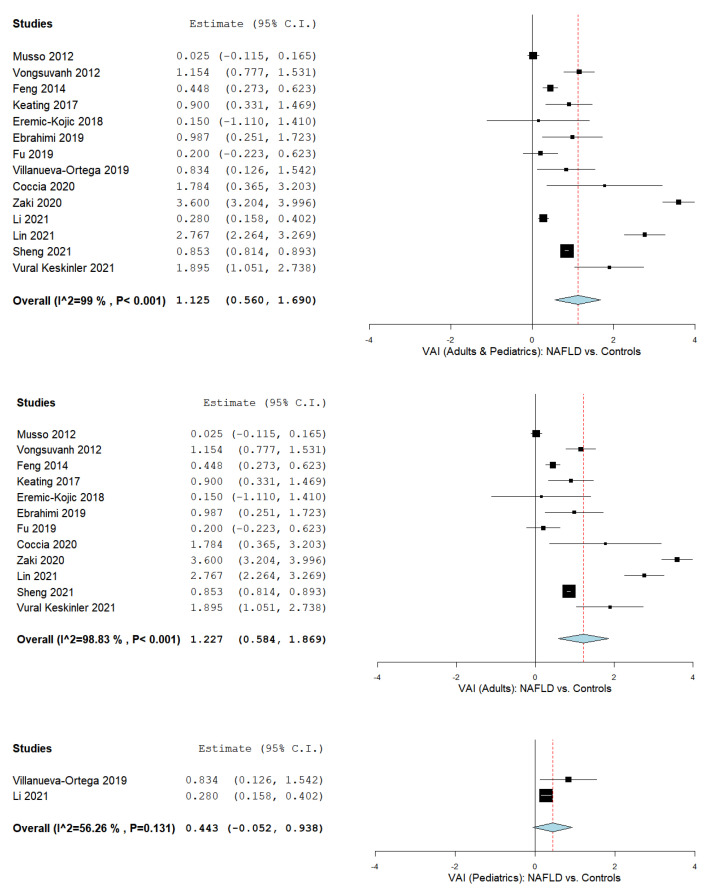
The VAI in NAFLD patients (adults and pediatrics) vs. controls, NAFLD patients (adults) vs. controls, and NAFLD patients (pediatrics) vs. controls.

The VAI was evaluated in a total of fourteen studies comparing values in NAFLD patients with control subjects [44,65,67,68,70,71,74,76,77,78,80,81,82,83]. Figure 2 summarizes the obtained meta-analysis results. The pooled analysis of included studies that assess the VAI in adult and pediatric NAFLD patients vs. control subjects showed an overall MD of 1.125 (95% CI 0.560–1.690). Considerable heterogeneity was reported with an I^2^ = 99% and a *p*-value < 0.001. Moreover, subgroup analysis was conducted in adults [44,65,67,68,70,71,74,77,78,80,82,83] and pediatrics [76,81] separately, with an MD of 1.1227 (95% CI 0.584–1.869) with considerable heterogeneity (I^2^ = 98.83% and a *p*-value < 0.001), and an MD of 0.443 (95% CI -0.052–0.938) with substantial heterogeneity (I^2^ = 56.26% and a *p*-value = 0.131), respectively.

**Figure 3:**
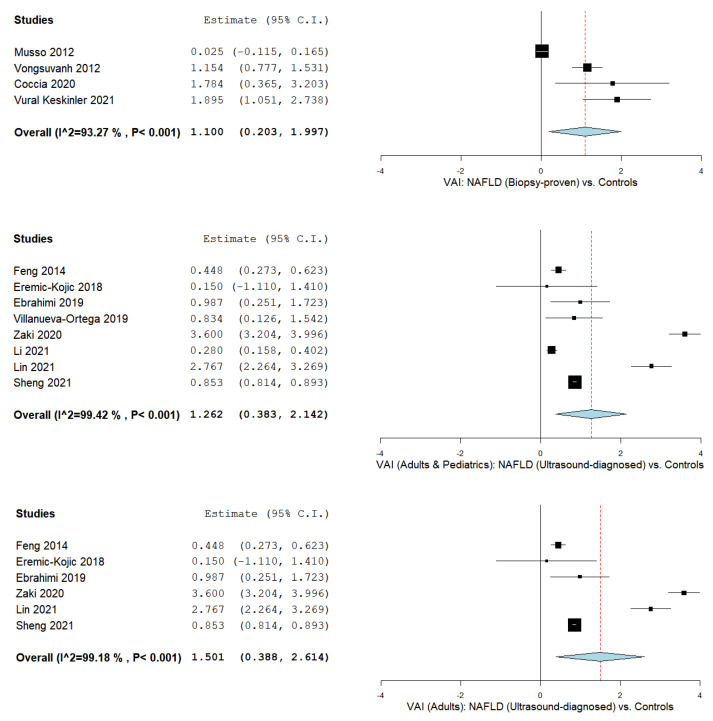
The VAI in biopsy-proven NAFLD vs. controls, ultrasound-diagnosed NAFLD (adults and pediatrics) vs. controls, and ultrasound-diagnosed NAFLD (adults) vs. controls.

We also evaluated the VAI in NAFLD patients vs. controls according to the hepatic steatosis diagnosis method as outlined in Figure 3, including a liver biopsy [44,65,78,82] in four studies with an MD of 1.100 (95% CI 0.203–1.997) with considerable heterogeneity (I^2^ = 93.27% and a *p*-value < 0.001), and ultrasonography in adult and pediatric NAFLD patients [67,68,70,76,77,80,81,83] in eight studies with an MD of 1.262 (95% CI 0.383–2.142) with considerable heterogeneity (I^2^ = 98.83% and a *p*-value < 0.001), as well as for adult NAFLD patients [67,68,70,77,80,83] in six studies with an MD of 1.501 (95% CI 0.388–2.614) with considerable heterogeneity (I^2^ = 99.18% and a *p*-value < 0.001).

### 3.5. The VAI and NASH

**Figure 4:**
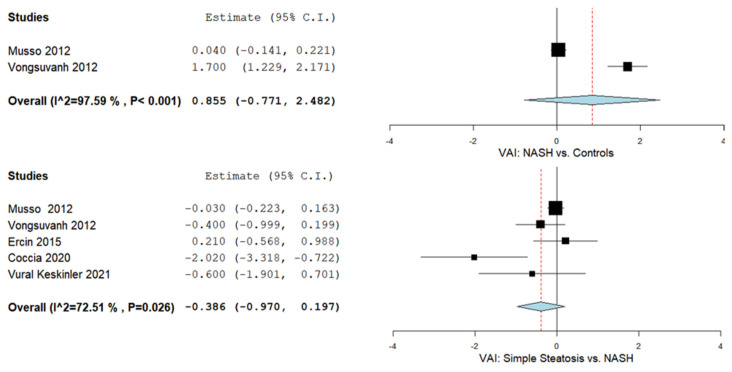
The VAI in NASH patients vs. controls and simple steatosis vs. NASH patients.

The VAI was evaluated in two studies comparing values in NAFLD patients with control subjects [44,78]. Figure 4 summarizes the obtained meta-analysis results. The pooled analysis of included studies assessing the VAI in NASH patients vs. control subjects showed an overall MD of 0.855 (95% CI—0.771–2.482) [44,78]. Considerable heterogeneity was reported with an I^2^ = 97.59% and a *p*-value < 0.001. Moreover, we evaluated the VAI in simple steatosis vs. NASH patients in five studies reporting an overall MD of −0.386 (95% CI—0.970–0.197) with substantial heterogeneity (I^2^ = 72.51% and a *p*-value = 0.026) [36,44,65,78,82].

### 3.6. The VAI and Hepatic Steatosis Severity

**Figure 5:**
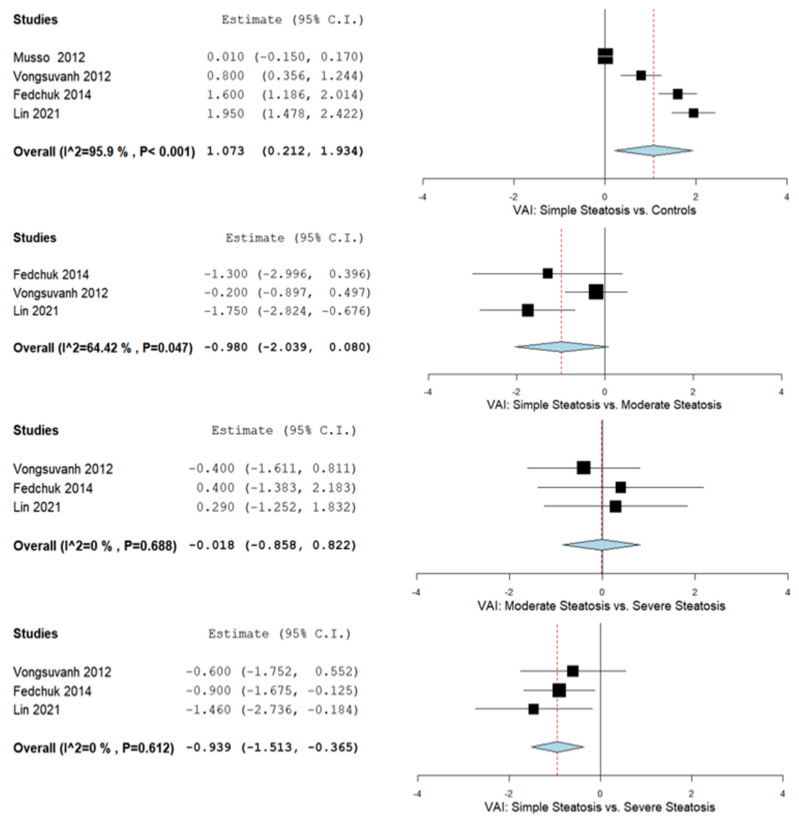
The VAI in simple steatosis vs. controls, simple steatosis vs. moderate steatosis, moderate steatosis vs. severe steatosis, and simple steatosis vs. severe steatosis.

A total of four studies compared VAI values in simple steatosis with control subjects [44,69,77,78]. Moreover, simple vs. moderate, moderate vs. severe, and simple vs. severe hepatic steatosis were evaluated in three studies [44,69,77] as outlined in Figure 5, summarizing the obtained meta-analysis results. The pooled analysis of included studies assessing VAI in simple steatosis patients vs. control subjects showed an overall MD of 1.073 (95% CI 0.212–1.934). Considerable heterogeneity was reported with an I^2^ = 95.9% and a *p*-value < 0.001. Furthermore, we evaluated the VAI in simple steatosis vs. moderate steatosis reporting an overall MD of −0.980 (95% CI—2.039–0.080) with considerable heterogeneity (I^2^ = 64.42% and a *p*-value = 0.047), moderate steatosis vs. severe steatosis reporting an overall MD of −0.018 (95% CI—0.858–0.822) without important heterogeneity (I^2^ = 0% and a *p*-value = 0.688), and simple steatosis vs. severe steatosis with a MD of −0.939 (95% CI—1.513–0.365) without important heterogeneity (I^2^ = 0% and a *p*-value = 0.612).

### 3.7. The VAI and Liver Fibrosis

**Figure 6:**
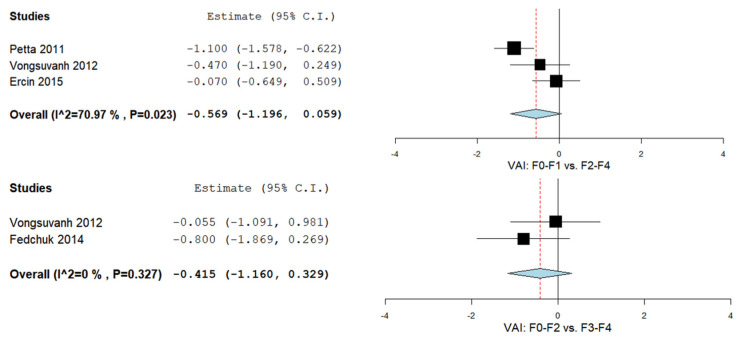
The VAI in liver fibrosis—F0–F1 vs. F2–F4 and F0–F2 vs. F3–F4.

The VAI was evaluated in a total of three studies comparing F0–F1 vs. F2–F4 [36,42,44] and another two comparing F0–F2 vs. F3–F4 [44,69]. Figure 6 summarizes the obtained meta-analysis results. The pooled analysis of included studies assessing VAI in F0–F1 vs. F2–F4 showed an overall MD of −0.569 (95% CI—1.196–0.059). Considerable heterogeneity was reported with an I^2^ = 70.97% and a *p*-value = 0.023. Moreover, a pooled analysis of included studies evaluating the VAI in F0–F2 vs. F3–F4 showed an overall MD of −0.415 (95% CI—1.160–0.329). No important heterogeneity was reported with an I^2^ = 0% and a *p*-value = 0.327.

### 3.8. The VAI and Diabetic/Prediabetic NAFLD Patients

**Figure 7:**
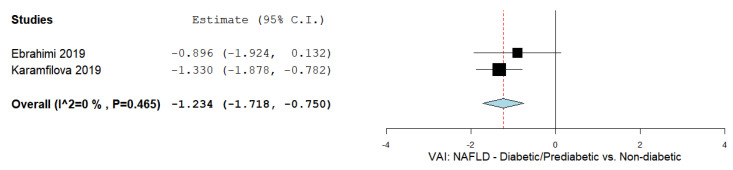
The VAI in type 2 diabetes mellitus/prediabetic NAFLD patients vs. non-diabetic NAFLD patients.

A total of two studies reported values of the VAI in diabetic and prediabetic patients with NAFLD [67,73]. Figure 7 summarizes the obtained meta-analysis results. The pooled analysis of included studies assessing the VAI in diabetic/prediabetic NAFLD patients showed an overall MD of −1.234 (95% CI—1.718– −0.750). No important heterogeneity was reported with an I^2^ = 0% and a *p*-value = 0.465.

### 3.9. The VAI and Males and Females

**Figure 8:**
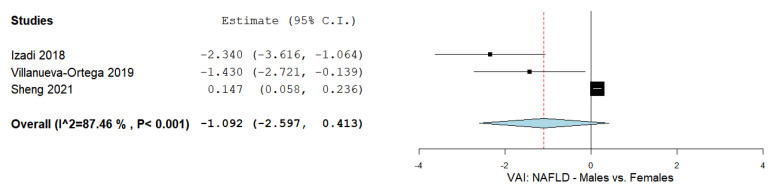
VAI in male vs. female NAFLD patients.

Two studies reported values of the VAI in male and female NAFLD patients [72,80,81]. Figure 8 summarizes the obtained meta-analysis results. The pooled analysis of included studies assessing the VAI in male and female NAFLD patients showed an overall MD of −1.092 (95% CI—2.597–0.413). Substantial heterogeneity was reported with an I^2^ = 87.46% and a *p*-value < 0.001.

### 3.10. VAI in Predicting NAFLD and NASH

**Figure 9:**
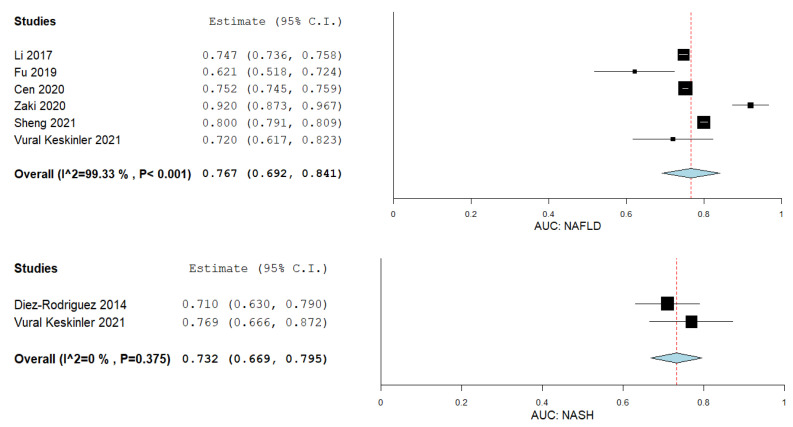
Accuracy of the VAI in predicting NAFLD and NASH.

A total of six studies evaluated the VAI in predicting NAFLD and two studies in predicting NASH. Figure 9 summarizes the results obtained in the meta-analysis. The pooled studies for the analysis evaluating the VAI in predicting NAFLD demonstrated an overall AUC of 0.767 with a 95% CI of 0.692–0.841, I^2^ = 99.33, and *p*-value < 0.001 [64,71,75,80,82,83]. VAI predicted NASH with an overall AUC of 0.732 with a 95% CI of 0.669–0.795, I^2^ = 0, and *p*-value of 0.375 [66,82].

### 3.11. Publication Bias, Sensitivity Analyses, and Meta-Regression

We performed several analyses, but only two analyses had more than 10 items to allow for a formal study of publication bias, sensitivity analysis, and meta-regression.

Supplementary Figure S1 outlines the results of the funnel plot for the VAI, comparing adult and pediatric NAFLD patients with controls, reporting the results of the publication bias test of *p* = 0.696. Alongside it, Supplementary Figure S2 outlines the results of the funnel plot for the VAI, comparing only adult NAFLD patients with controls, reporting the results of the publication bias test of *p* = 0.565.

Since the results had high heterogeneity in all subjects (adults and children group), as well as in the children-only group comparing NASH with controls regarding the means of the VAI, we performed a leave-one-out analysis (Supplementary Figures S3 and S4). No matter what study was removed, the results remained statistically significant, but the heterogeneity remain high too (I^2^ above 95%). Although there were several outliers (some diminishing the MD—Musso and Li for the adults and children group [76,78]—and two increasing the MD—Zaki and Lin [77,83]), only Zaki was an influential point, in both meta-analyses [83]. Nevertheless, the heterogeneity after removing it did not diminish greatly (96% or 95%), and the result remained statistically significant, MD = 0.98 (95% CI 0.48–1.49), and preserved the same direction. Moreover, we tried to exclude both the Zaki and Lin studies to see if the result was robust, and it was: MD = 0.97 (95% CI 0.51–1.43)—for all subjects, I^2^ = 97.4% (*p* < 0.001); MD = 1 (95% CI 0.33–1.68)—for children-only group, *p* = 0.004, maintaining the same direction and being statistically significant, I^2^ = 97.4% (*p* < 0.001). The removal of the Musso or Li studies would have given more weight to the observed difference and thus sustain our findings. In conclusion, the heterogeneity is high, but the results seem robust.

In order to check the source of the unaccounted heterogeneity, we built several metaregression models adjusting for publication year, risk of bias in patient selection, risk of bias in standard test quality, and a multivariate model including the publication year and patient selection quality for studies on adults and children, as well as for studies on adults only (Supplementary Tables S3 and S4). The patient risk of bias in selection quality and the risk of bias in standard test quality were selected for metaregression after observation using the QUADAS-2 tool while assessing the risk of bias, as these were the domains of the tool with the highest variability (the other domains were without bias or rarely with biased). We did not find any statistically significant associations. Nevertheless, the more recent the publication year of the study the bigger the mean difference of the VAI between NASH and controls, in both univariate and multivariate models in studies on children as well as in the multivariate model in studies on adults and children, being closer to the significance level (*p*-values below 0.11). The higher the risk of bias concerning the standard test (studies not using a liver biopsy), the higher the mean difference of the VAI between NASH and controls, especially in the multivariate model, but not reaching the significance level.

### 3.12. Bias Evaluation

The risk of bias in individual studies was evaluated using the QUADAS-2 tool as outlined in Supplementary Figure S5 and Table S2. There were several issues regarding the bias present in the reviewed studies: about 60% of studies did not use the same reference test for all subjects included in their study; several studies did not have a complete description of how the reference test was performed; and information regarding the biopsy sample details and histological evaluation was not completely described in all studies that performed a liver biopsy. Therefore, the reference standard, its conduct, or its interpretation could have introduced bias. Furthermore, almost half of the studies had a high risk of bias regarding patient selection. Moreover, in 80% of the studies, the included patients might not match the review questions, posing a problem regarding their applicability. It was not clear if the reference standard results were interpreted without knowledge of the results of the index test in all the assessed studies, but this is highly unlikely to have been the case. Likewise, 20% of the studies had unclear applicability, raising concern that the index test, its conduct, or interpretation might differ from the review question. Moreover, the test operators’ training and the withholding of an eventual treatment during the diagnostic tests were not specified in most studies.

## 4. Discussion

Lately, several scores and biomarkers have been studied in order to avoid a liver biopsy, an invasive procedure that is currently the current gold standard with which to diagnose NAFLD and liver fibrosis. In our systematic review and meta-analysis, we evaluated VAI values in NAFLD according to diagnosis method, sex, and the presence of T2DM, in addition to NASH, quantifying liver fibrosis and hepatic steatosis severity. We also assessed the accuracy of the VAI in predicting NAFLD and NASH. We included twenty-four articles with a total population of 70,519 in our qualitative synthesis, out of which 22 studies were included in our quantitative synthesis. We reported that the VAI is significantly increased in adult NAFLD patients, whether biopsy-proven or ultrasound-diagnosed, in contrast with the pediatric population, where there was no significant association between the VAI and NAFLD. Additionally, the VAI was able to discriminate between simple steatosis and controls, as well as severe steatosis and simple steatosis. Diabetic or prediabetic NAFLD patients presented significantly different VAI values compared to NAFLD non-diabetic patients. However, it was not able to differentiate between NASH and controls, NASH and simple steatosis, liver fibrosis severity, simple and moderate, as well as moderate and severe steatosis. Furthermore, no significantly different VAI values were reported between sexes in NAFLD patients. Compared to other studied noninvasive markers, our results showed that the VAI has a good predictive value in diagnosing NAFLD and NASH.

It is worthwhile to highlight that a consensus of experts suggested changing the name NAFLD to metabolic-dysfunction-associated fatty liver disease (MAFLD), a shift in the paradigm and the underlying pathogenesis towards a more general term that does not specifically address NAFLD. The diagnostic criteria of MAFLD consists of hepatic steatosis combined with one of the following three elements: overweight/obesity, the presence of T2DM, and evidence of metabolic dysregulation, which is different from NAFLD; therefore, our study results reflect findings associated with NAFLD solely [84,85].

We reported several results that need to be further developed. Firstly, the prevalence of NAFLD in our study was 36%, with a gender distribution of almost 60% males and 40% females. The latter can be explained by the fact that some studies were male exclusive. Our study involved populations from diverse ethnicities and backgrounds, making our results more generalizable and reliable. The current guidelines recommend using a liver biopsy for the diagnosis and grading of NAFLD, NASH, and liver fibrosis [15]. However, most included studies in our review used ultrasonography, while only five used liver biopsies.

The mechanism explaining the relationship between the VAI and NAFLD is simple. In the study conducted by Kim et al., the authors reported that larger areas of visceral adipose tissue were longitudinally associated with a higher risk of incident NAFLD over 4 years, and that the distribution of fat exerts greater effects on NAFLD than the content itself. VAI is a simple and easy-to-calculate indirect marker of visceral adipose tissue based on BMI, WC, triglycerides, and HDL, parameters that describe and relate to adipose tissue dysfunction maldistribution [86].

We reported a significant increase in VAI values in NAFLD (biopsy-proven and ultrasound-diagnosed) adult patients. However, this association was not found in pediatric NAFLD patients. Al-Daghri et al. found that BMI was superior to the VAI, describing the association with insulin resistance, adipokines, and subclinical inflammation [87]. In fact, this could be explained by the fact that visceral adipose tissue and inflammation markers do not correlate with insulin sensitivity in children [88]. Furthermore, the current version of the VAI fails to factor in the physiological changes that the pediatric population go through; the use of the VAI on children equates to considering them as “small adults”. The numerical constants used in the VAI mathematical formula are based on the healthy Caucasian adult population. Amato et al. (designers of the VAI) state that the index should not be used in non-Caucasian and pediatric populations in its current version [89]. In an effort to remediate this, Hernández et al. designed the pediatric metabolic index (PMI), which correlates with the homeostatic model assessment of insulin resistance (HOMA-IR), the Matsuda insulin resistance index, and transaminases. This novel index should be further evaluated [90].

Moreover, we found that VAI values were significantly higher in patients with simple steatosis compared with controls and in severe steatosis compared with simple steatosis. However, we were not able to find any significant mean differences between simple and moderate steatosis, as well as moderate and severe steatosis. This can be attributed to the fact that VAI is an indirect surrogate marker for visceral adiposity and does not directly reflect the process involved in hepatic steatosis, fibrosis, and NASH. A study conducted by Fedchuk et al. included the VAI, the FLI, the NAFLD liver fat score (NAFLD-LFS), the hepatic steatosis index (HSI), and the triglyceride-glucose index (TryG). The outcome was that aside from the VAI, all markers showed a linear trend with histological steatosis grading, albeit with a weak-to-moderate correlation with the histological amount of liver steatosis. Only the FLI and NAFLD indexes were able to discriminate between moderate and mild steatosis. However, none of these indexes were able to distinguish between moderate and severe steatosis. In detecting the highest grade of steatosis all did poorly, with the NAFLD-LFS having the highest AUROC (0.72). All markers were confirmed to lack the ability to predict more severe steatosis grades vs. no/mild in comparison to ultrasound as well, with the FLI having the highest AUROC (0.69) [69].

In our meta-analysis, we found no significant MD in VAI values related to liver fibrosis severity. However, other biomarkers were found to be related to fibrosis severity, including the NFS with an AUROC of 0.81–0.88, which was able to predict advanced fibrosis in 77% and exclude significant fibrosis in 92.5%. FibroTest, which is a combination of serum biomarkers, had an AUROC of 0.86 in predicting F2 to F4, and was more performant in detecting F3–F4. Similarly, a meta-analysis conducted by Vali et al. showed that FibroTest had an acceptable diagnostic performance, AUC > 0.80, only in detecting cirrhosis, demonstrated by the test’s high negative predictive value (NPV) of 0.90 and 0.98, value for advanced fibrosis in other chronic liver diseases, and was better used to rule out advanced liver fibrosis [91,92]. The fatty liver index (FLI) had an AUROC of 0.85. FIB-4 was designed to predict advanced fibrosis in patients with HCV and HIV coinfection, where the index had an AUROC of 0.86. Moreover, the aspartate aminotransferase (AST)-to-platelet ratio was found to be best used in ruling out advanced fibrosis, with an AUROC that varied depending on the studies in a range from 0.67 to 085. Likewise, the BARD FIB-4’s score performance varied depending on the studies, ranging from 0.67–0.865, and was found to be better in ruling out advanced fibrosis and cirrhosis as well in combination with another noninvasive fibrosis models. The enhanced liver fibrosis (ELF) panel of multiple biomarkers was found to have an AUROC of 0.90, the highest among the indices, in predicting advanced fibrosis F3 and F4. However, it had a lower AUROC (0.76) in predicting F2 to F4 diagnosis [91].

We reported the accuracy of the VAI in predicting NAFLD, with an AUC of 0.767. In comparison to the VAI, the accuracy of other noninvasive scores in predicting NAFLD has been evaluated, including the FLI and HSI, which had an AUROC of 0.84 and 0.81, respectively. Moreover, the NAFLD-LFS was reported to have an AUROC of 0.86–0.87. SteatoTest, a noninvasive score composed of a panel of specialized tests, was found to have an AUROC of 0.79–0.80 in predicting biopsy-proven hepatic steatosis [93].

Although we found no significant difference in VAI values between NASH patients and controls, as well as NASH patients and simple steatosis, we were able to assess its accuracy in predicting NASH, reporting an AUC of 0.732. A recent meta-analysis conducted by Ismaiel et al. reported that FIB-4 (AUC 0.729) was better in predicting NASH than the NAFLD fibrosis score (NFS) (AUC 0.687) [94]. Moreover, Lee et al. evaluated cytokeratin 18 (CK-18), an intermediate filament released upon hepatocytes’ death, in predicting NASH. The authors reported an AUC of 0.82 for CK-19 (M65), while CK-18 (M30) had an AUC of 0.75 [93]. Thus, VAI performed poorly only in comparison to CK-18 (M65 and M30) in predicting NASH. Conversely, the shortcomings of CK-18 impede its roll-out and usage in clinical settings, and those include its commercial unavailability, low sensitivity, and variability in suggested cut-offs and diagnostic accuracy among studies [95].

There were no signs of publication bias, nor in the funnel plot, nor in the formal statistical test for publication bias.

Several meta-analyses had high heterogeneity, but only two allowed a closer inspection of its effect and possible causes, having a sufficient number of included studies. When comparing NASH with controls regarding the means of the VAI in all subjects (adults and children group), as well as in the children-only group, the results were robust after the exclusion of influential studies and outliers, and while performing the leave-one-out analyses. The results remained statistically significant, with minor clinical differences, and also, the direction of the result did not change; thus the analyzed results are robust. Nevertheless, the heterogeneity remained high, even after performing these procedures. Furthermore, we used metaregression in order to identify the possible sources for the unaccounted heterogeneity. The results were not statistically significant, which is common since metaregressions are often underpowered (due to the small number of included studies). Nevertheless, there was a tendency for more important differences in mean VAI between NASH and control groups in newer studies, possibly due to better-designed studies. Additionally, there was a tendency for smaller observed differences in studies not using a liver biopsy, suggesting that noninvasive biomarker identification of NASH overestimates the diagnostic accuracy of the VAI.

Some important limitations in our systematic review and meta-analysis should be mentioned. Due to the observational design of the included studies, causality cannot be inferred between the VAI and NAFLD, NASH, hepatic steatosis, or liver fibrosis. While most studies used ultrasonography instead of a liver biopsy, it is possible that the prevalence of NAFLD is underestimated. Moreover, only two studies reported on the VAI in pediatric NAFLD patients. Besides, the current literature is very limited in data evaluating the VAI in NASH and liver fibrosis. Therefore, more studies evaluating the VAI in pediatric NAFLD patients, NASH, and liver fibrosis are necessary. As NAFLD is associated with insulin resistance, studies on the VAI in diabetic and non-diabetic NAFLD patients are of great relevance. However, only two studies provided VAI values according to the presence or absence of diabetes/prediabetes. Very few studies reported on how the biopsy was performed or the experience of the pathologists, which casts some doubts on the accuracy of the performed reference test. The main limitation of the currently available noninvasive scores is the absence of a consensus on the cut-off point and ideal threshold that can balance between sensitivity and specificity. Another significant limitation is that 60% of the studies did not use the same test for all the subjects included in the study; usually the case group received a biopsy, while the controls received other (less sensitive) tests. Moreover, information regarding the handling of a biopsy, histological assessment, and test operators’ training were lacking, and information about the reduction in diagnostic ability of the standard test might be missing. Both previously reported issues can induce a directional classification bias, diminishing the accuracy of the results, more likely by underestimating the presence or severity of the liver disease. The patient selection had a high risk of bias in almost half of the studies, mostly due to the use of a case–control design and the absence of reporting of consecutive sampling, which might diminish the accuracy and generalizability of results. Overall, the biases tend to overestimate the diagnosis ability of the index, a situation that is common to studies reporting similar indices since they commonly suffer from the same problems (reduced use of liver biopsy, case–control designs). Some of the subgroup analyses in our review contained a small number of studies, indicating underpowered analyses and a reduced robustness of those results, which should be kept in mind. Nevertheless, the main result of our meta-analysis is based on an important number of studies and many subjects (e.g., 14 studies and 20688 subjects).

Nevertheless, our systematic review and meta-analysis have important strengths. The theme of this review is of great clinical significance, as the rapid increase in NAFLD prevalence and associated complications call for an urgent need for an easy and simple noninvasive method that could be used in clinical settings in order to screen these patients. We provided data regarding the accuracy of the VAI in predicting NAFLD and NASH that can be compared to other noninvasive markers and scores reported in published studies. We believe that our review sheds light on the missing data in the current literature that needs further assessment in future studies, while summarizing the current literature in a nonbiased manner. Moreover, our search strategy was comprehensive and comprised of several medical databases, which allowed us to study the association in a systematic manner from several ethnicities, regions, and backgrounds, allowing us to have more generalizable results.

## 5. Conclusions

In conclusion, we found that the VAI is significantly increased in adult but not pediatric NAFLD patients, while being able to discriminate between simple steatosis and controls as well as severe steatosis and simple steatosis. Nevertheless, no significant mean difference in VAI values was found between NASH and controls, NASH and simple steatosis, liver fibrosis severity, simple and moderate, as well as moderate and severe steatosis. The reported accuracy of the VAI in predicting NAFLD was considered acceptable compared to other currently recommended scores and markers. Due to limited published data regarding the VAI in NASH patients, pediatric populations with NAFLD, liver fibrosis, and diabetic patients with NAFLD, future research is deemed necessary.

## Data Availability

Not applicable. The analysed data was extracted from the cited original articles, the quality assessment data is published in the Supplementary File.

## Supplementary Materials

The following are available online at https://www.mdpi.com/article/10.3390/biomedicines9121890/s1, Supplementary material: Search Strategy, Supplementary Table S1: The visceral adiposity index in NAFLD; Supplementary Table S2: QUADAS-2 tool for evaluating the methodological quality of included studies; Supplementary Table S3: Meta-regression models adjusting for publication year, patient selection quality, and standard quality, and a multivariate model including the publication year and patient selection bias risk, for studies on adults; Supplementary Table S4: Meta-regression models adjusting for publication year, patient selection quality, and standard quality, and a multivariate model including the publication year and patient selection bias risk, for studies on adults and children; Supplementary Figure S1: Funnel plot for the VAI, comparing adult and pediatric NAFLD patients with controls, with publication bias test (*p*-value = 0.696); Supplementary Figure S2: Funnel plot for the VAI, comparing adult NAFLD patients with controls, with publication bias test (*p*-value = 0. 565); Supplementary Figure S3: Leave-one-out analysis for studies on adults and pediatrics showing the mean difference and I^2^ along with 95% confidence intervals; Supplementary Figure S4: Leave-one-out analysis for studies on adults showing the mean difference and I^2^ along with 95% confidence intervals; Supplementary Figure S5: Summary of the QUADAS-2 quality assessment according to the risk of bias and applicability of included studies.

## References

[1] Sporea I., Popescu A., Dumitrascu D., Brisc C., Nedelcu L., Trifan A., Gheorghe L., Fierbinteanu Braticevici C. (2018). Nonalcoholic Fatty Liver Disease: Status Quo. J. Gastrointestin. Liver Dis..

[2] Younossi Z.M., Koenig A.B., Abdelatif D., Fazel Y., Henry L., Wymer M. (2016). Global epidemiology of nonalcoholic fatty liver disease—Meta-analytic assessment of prevalence, incidence, and outcomes. Hepatology.

[3] Younossi Z., Anstee Q.M., Marietti M., Hardy T., Henry L., Eslam M., George J., Bugianesi E. (2018). Global burden of NAFLD and NASH: Trends, predictions, risk factors and prevention. Nat. Rev. Gastroenterol. Hepatol..

[4] Söderberg C., Stål P., Askling J., Glaumann H., Lindberg G., Marmur J., Hultcrantz R. (2010). Decreased survival of subjects with elevated liver function tests during a 28-year follow-up. Hepatology.

[5] Ekstedt M., Franzén L.E., Mathiesen U.L., Thorelius L., Holmqvist M., Bodemar G., Kechagias S. (2006). Long-term follow-up of patients with NAFLD and elevated liver enzymes. Hepatology.

[6] Angulo P. (2002). Nonalcoholic Fatty Liver Disease. N. Engl. J. Med..

[7] Musso G., Gambino R., Cassader M., Pagano G. (2011). Meta-analysis: Natural history of non-alcoholic fatty liver disease (NAFLD) and diagnostic accuracy of noninvasive tests for liver disease severity. Ann. Med..

[8] Musso G., Gambino R., Tabibian J.H., Ekstedt M., Kechagias S., Hamaguchi M., Hultcrantz R., Hagström H., Yoon S.K., Charatcharoenwitthaya P. (2014). Association of Non-alcoholic Fatty Liver Disease with Chronic Kidney Disease: A Systematic Review and Meta-analysis. PLoS Med..

[9] FAN J.-G., ZHU J., LI X.-J., CHEN L., LU Y.-S., LI L., DAI F., LI F., CHEN S.-Y. (2005). Fatty liver and the metabolic syndrome among Shanghai adults. J. Gastroenterol. Hepatol..

[10] Yang K.C., Hung H.-F., Lu C.-W., Chang H.-H., Lee L.-T., Huang K.-C. (2016). Association of Non-alcoholic Fatty Liver Disease with Metabolic Syndrome Independently of Central Obesity and Insulin Resistance. Sci. Rep..

[11] Vanni E., Bugianesi E., Kotronen A., De Minicis S., Yki-Järvinen H., Svegliati-Baroni G. (2010). From the metabolic syndrome to NAFLD or *vice versa*?. Dig. Liver Dis..

[12] Sung H.H., Park C.E., Gi M.Y., Cha J.A., Moon A.E., Kang J.K., Seong J.M., Lee J.H., Yoon H. (2020). The association of the visceral adiposity index with insulin resistance and beta-cell function in Korean adults with and without type 2 diabetes mellitus. Endocr. J..

[13] Amato M.C., Pizzolanti G., Torregrossa V., Misiano G., Milano S., Giordano C. (2014). Visceral Adiposity Index (VAI) Is Predictive of an Altered Adipokine Profile in Patients with Type 2 Diabetes. PLoS ONE.

[14] (2009). Visceral Fat and Adiponectin: Associations with Insulin Resistance Are Tissue-Specific in Women. Metab. Syndr. Relat. Disord..

[15] Ando Y., Jou J.H. (2021). Nonalcoholic Fatty Liver Disease and Recent Guideline Updates. Clin. Liver Dis..

[16] Actis G.C., Olivero A., Lagget M., Pellicano R., Smedile A., Rizzetto M. (2007). The Practice of Percutaneous Liver Biopsy in a Gastrohepatology Day Hospital: A Retrospective Study on 835 Biopsies. Dig. Dis. Sci..

[17] Monelli F., Venturelli F., Bonilauri L., Manicardi E., Manicardi V., Rossi P.G., Massari M., Ligabue G., Riva N., Schianchi S. (2021). Systematic review of existing guidelines for NAFLD assessment. Hepatoma Res..

[18] Shen J., Chan H.L.-Y., Wong G.L.-H., Chan A.W.-H., Choi P.C.-L., Chan H.-Y., Chim A.M.-L., Yeung D.K.-W., Yu J., Chu W.C.-W. (2012). Assessment of non-alcoholic fatty liver disease using serum total cell death and apoptosis markers. Aliment. Pharmacol. Ther..

[19] Chan W.-K., Sthaneshwar P., Nik Mustapha N.R., Mahadeva S. (2014). Limited Utility of Plasma M30 in Discriminating Non-Alcoholic Steatohepatitis from Steatosis—A Comparison with Routine Biochemical Markers. PLoS ONE.

[20] Leoni S., Tovoli F., Napoli L., Serio I., Ferri S., Bolondi L. (2018). Current guidelines for the management of non-alcoholic fatty liver disease: A systematic review with comparative analysis. World J. Gastroenterol..

[21] Amato M.C., Giordano C., Galia M., Criscimanna A., Vitabile S., Midiri M., Galluzzo A., for the AlkaMeSy Study Group (2010). Visceral Adiposity Index: A reliable indicator of visceral fat function associated with cardiometabolic risk. Diabetes Care.

[22] Kouli G.M., Panagiotakos D.B., Kyrou I., Georgousopoulou E.N., Chrysohoou C., Tsigos C., Tousoulis D., Pitsavos C. (2017). Visceral adiposity index and 10-year cardiovascular disease incidence: The ATTICA study. Nutr. Metab. Cardiovasc. Dis..

[23] Bijari M., Jangjoo S., Emami N., Raji S., Mottaghi M., Moallem R., Jangjoo A., Saberi A. (2021). The Accuracy of Visceral Adiposity Index for the Screening of Metabolic Syndrome: A Systematic Review and Meta-Analysis. Int. J. Endocrinol..

[24] Page M.J., McKenzie J.E., Bossuyt P.M., Boutron I., Hoffmann T.C., Mulrow C.D., Shamseer L., Tetzlaff J.M., Akl E.A., Brennan S.E. (2021). The PRISMA 2020 statement: An updated guideline for reporting systematic reviews. BMJ.

[25] Ismaiel A., Jaaouani A., Leucuta D.-C., Popa S.-L., Dumitrascu D.L. (2021). The Visceral Adiposity Index in Non-Alcoholic Fatty Liver Disease and Liver Fibrosis—Systematic Review and Meta Analysis. Inplasy protocol.

[26] Whiting P.F., Rutjes A.W.S., Westwood M.E., Mallett S., Deeks J.J., Reitsma J.B., Leeflang M.M.G., Sterne J.A.C., Bossuyt P.M.M., The QUADAS-2 Group (2011). QUADAS-2: A Revised Tool for the Quality Assessment of Diagnostic Accuracy Studies. Ann. Intern. Med..

[27] Wallace B.C., Dahabreh I.J., Trikalinos T.A., Lau J., Trow P., Schmid C.H. (2012). Closing the Gap between Methodologists and End-Users: R as a Computational Back-End. J. Stat. Softw..

[28] Viechtbauer W. (2010). Conducting Meta-Analyses in R with the metafor Package. J. Stat. Softw..

[29] Robin X., Turck N., Hainard A., Tiberti N., Lisacek F., Sanchez J.-C., Müller M. (2011). pROC: An open-source package for R and S+ to analyze and compare ROC curves. BMC Bioinform..

[30] Obuchowski N.A., Lieber M.L., Wians F.H. (2004). ROC Curves in Clinical Chemistry: Uses, Misuses, and Possible Solutions. Clin. Chem..

[31] Harrer M., Cuijpers P., Furukawa T., Ebert D.D. (2019). Dmetar: Companion R Package for The Guide ‘Doing Meta-Analysis in R’. https://dmetar.protectlab.org/.

[32] R Core Team (2020). R: A Language and Environment for Statistical Computing.

[33] Balduzzi S., Rücker G., Schwarzer G. (2019). How to perform a meta-analysis with R: A practical tutorial. Evid. Based Ment. Health.

[34] Dynnyk N., Svintsitsky A., Solovyova G., Bogomaz V., Baka O., Gurbych O., Golovchanska Y. (2016). Physical activity reduce hepatic apoptosis in patients with non-alcoholic fatty liver disease and visceral obesity. J. Hepatol..

[35] Elsaid M., Li Y., John T., Catalano C., Rustgi V.K. (2019). Racial and gender disparities in the relationship between non-alcoholic fatty liver disease and visceral adipose dysfunction. Hepatology.

[36] Ercin C.N., Dogru T., Genc H., Celebi G., Aslan F., Gurel H., Kara M., Sertoglu E., Tapan S., Bagci S. (2015). Insulin resistance but not visceral adiposity index is associated with liver fibrosis in nondiabetic subjects with nonalcoholic fatty liver disease. Hepatol. Int..

[37] Ercin C.N., Dogru T., Tapan S., Genc H., Aslan F., Çelebi G., Kara M., Sertoglu E., Karslioglu Y., Kurt I. (2013). Visceral adiposity index in nonalcoholic fatty liver disease: Association with hepatic and systemic inflammation. Biochim. Clin..

[38] Keating S., Parker H., Hickman I., Wallen M., George J., Johnson N. (2017). Can equation-based indices be used to detect longitudinal change in 1h-MRS quantified intra-hepatic lipid in clinical practice?. Inflamm. Intest. Dis..

[39] Kondo T., Kitano S., Miyakawa N., Watanabe T., Goto R., Sakaguchi M., Igata M., Kawashima J., Motoshima H., Matsumura T. (2020). Activation of heat shock response ameliorates nonalcoholic fatty liver disease biomarkers. Diabetes.

[40] Kouvari M., Panagiotakos D., Chrysohoou C., Georgousopoulou E., Tousoulis D., Pitsavos C. (2020). Visceral adiposity index, non-alcoholic fatty liver disease and 10-year cardiovascular disease incidence: A gender-based analysis from the attica prospective (2002–2012) study. J. Am. Coll. Cardiol..

[41] Nascimbeni F., Fedchuk L., Pais R., Charlotte F., Housset C., Loria P., Ratziu V. (2014). Performance and limitations of five steatosis biomarkers in patients with Non-alcoholic Fatty Liver Disease. Hepatology.

[42] Petta S., Amato M., Di Marco V., Cammà C., Pizzolanti G., Rosa Barcellona M., Cabibi D., Galluzzo A., Sinagra D., Giordano C. (2011). Visceral adiposity index is associated with significant fibrosis in patients with nonalcoholic fatty liver disease. Hepatology.

[43] Petta S., Amato M., Licata G., Barcellona M., Cammà C., Cabibi D., Di Marco V., Giordano C., Sinagra D., Galluzzo A. (2011). Visceral adiposity index, expression of adipose dysfunction, is associated with significant fibrosis in patients with non-alcoholic fatty liver disease. Dig. Liver Dis..

[44] Vongsuvanh R., George J., Van Der Poorten D. (2011). Visceral adiposity index is not a predictor of liver histology in patients with nonalcoholic fatty liver disease. J. Gastroenterol. Hepatol..

[45] Filik L. (2012). Visceral adiposity index and exercise in non-alcoholic fatty liver disease. Aliment. Pharmacol. Ther..

[46] Li Y., Liu L., Wang B., Chen D. (2013). Letter: Is visceral adiposity index a predictor of liver histology in patients with non-alcoholic fatty liver disease?. Aliment. Pharmacol. Ther..

[47] Petta S., Craxì A. (2012). Visceral adiposity index and exercise in non-alcoholic fatty liver disease: Authors’ reply. Aliment. Pharmacol. Ther..

[48] Barrea L., Annunziata G., Muscogiuri G., Di Somma C., Laudisio D., Maisto M., de Alteriis G., Tenore G.C., Colao A., Savastano S. (2018). Trimethylamine-N-oxide (TMAO) as Novel Potential Biomarker of Early Predictors of Metabolic Syndrome. Nutrients.

[49] Ibarra-Reynoso L.D.R., Pisarchyk L., Pérez-Luque E.L., Garay-Sevilla M.E., Malacara J.M. (2014). Whole-body and hepatic insulin resistance in obese children. PLoS ONE.

[50] Loureiro L.M., Cordeiro A., Mendes R., Luna M., Pereira S., Saboya C.J., Ramalho A. (2019). Clinic, anthropometric and metabolic changes in adults with class III obesity classified as metabolically healthy and metabolically unhealthy. Diabetes Metab. Syndr. Obes. Targets Ther..

[51] Vassilatou E., Lafoyianni S., Vassiliadi D.A., Ioannidis D., Paschou S.A., Mizamtsidi M., Panagou M., Vryonidou A. (2018). Visceral adiposity index for the diagnosis of nonalcoholic fatty liver disease in premenopausal women with and without polycystic ovary syndrome. Maturitas.

[52] Reeds D.N., Chambers K.T., Patterson B.W., Finck B.N. (2017). Intrahepatic triglyceride is more strongly associated with insulin-resistant glucose metabolism than visceral adiposity in HIV, and is improved with tauroursodeoxycholic acid treatment. Antivir. Ther..

[53] Sterling R.K., King W.C., Khalili M., Kleiner D.E., Hinerman A.S., Sulkowski M., Chung R.T., Jain M.K., Lisker-Melman M., Wong D.K. (2021). Performance of Serum-Based Scores for Identification of Mild Hepatic Steatosis in HBV Mono-infected and HBV–HIV Co-infected Adults. Dig. Dis. Sci..

[54] Barrea L., Muscogiuri G., Modica R., Altieri B., Pugliese G., Minotta R., Faggiano A., Colao A., Savastano S. (2021). Cardio-Metabolic Indices and Metabolic Syndrome as Predictors of Clinical Severity of Gastroenteropancreatic Neuroendocrine Tumors. Front. Endocrinol..

[55] Xu C., Ma Z., Wang Y., Liu X., Tao L., Zheng D., Guo X., Yang X. (2018). Visceral adiposity index as a predictor of NAFLD: A prospective study with 4-year follow-up. Liver Int..

[56] Balducci S., Cardelli P., Pugliese L., D’Errico V., Haxhi J., Alessi E., Iacobini C., Menini S., Bollanti L., Conti F.G. (2015). Volume-dependent effect of supervised exercise training on fatty liver and visceral adiposity index in subjects with type 2 diabetes The Italian Diabetes Exercise Study (IDES). Diabetes Res. Clin. Pract..

[57] Della Pepa G., Russo M., Vitale M., Carli F., Vetrani C., Masulli M., Riccardi G., Vaccaro O., Gastaldelli A., Rivellese A.A. (2021). Pioglitazone even at low dosage improves NAFLD in type 2 diabetes: Clinical and pathophysiological insights from a subgroup of the TOSCA.IT randomised trial. Diabetes Res. Clin. Pract..

[58] Ebrahimi S., Gargari B.P., Aliasghari F., Asjodi F., Izadi A. (2020). Ramadan fasting improves liver function and total cholesterol in patients with nonalcoholic fatty liver disease. Int. J. Vitam. Nutr. Res..

[59] Gelli C., Tarocchi M., Abenavoli L., Di Renzo L., Galli A., De Lorenzo A. (2017). Effect of a counseling-supported treatment with the Mediterranean diet and physical activity on the severity of the non-alcoholic fatty liver disease. World J. Gastroenterol..

[60] Montesi L., Caselli C., Centis E., Nuccitelli C., Moscatiello S., Suppini A., Marchesini G. (2014). Physical activity support or weight loss counseling for nonalcoholic fatty liver disease?. World J. Gastroenterol..

[61] Reginato E., Pippi R., Aiello C., Tomaro E.S., Ranucci C., Buratta L., Bini V., Marchesini G., De Feo P., Fanelli C. (2019). Effect of short term intensive lifestyle intervention on hepatic steatosis indexes in adults with obesity and/or type 2 diabetes. J. Clin. Med..

[62] Silva E.I.G., Guedes S.E.M., Cunha B.E.S., Tomiya M.T.O., da Silva A.M.D., de Brito C.A. (2019). Sociodemographic and nutritional parameters of carriers of non-alcoholic fatty liver disease. Acta Gastroenterol. Latinoam..

[63] Qi J., Lin Q., Lin X., Chen X. (2015). Relationship of visceral adiposity index with serum aminotransferase and nonalcoholic fatty liver disease in patients with sleep apnea. Zhonghua Yi Xue Za Zhi.

[64] Cen C., Wang W., Yu S., Tang X., Liu J., Liu Y., Zhou L., Yu J., Zheng S. (2020). Development and validation of a clinical and laboratory-based nomogram to predict nonalcoholic fatty liver disease. Hepatol. Int..

[65] Coccia F., Testa M., Guarisco G., Bonci E., Di Cristofano C., Silecchia G., Leonetti F., Gastaldelli A., Capoccia D. (2020). Noninvasive assessment of hepatic steatosis and fibrosis in patients with severe obesity. Endocrine.

[66] Díez-Rodríguez R., Ballesteros-Pomar M.D., Calleja-Fernández A., González-De-Francisco T., González-Herráez L., Calleja-Antolín S., Cano-Rodríguez I., Olcoz-Goñi J.L. (2014). Insulin resistance and metabolic syndrome are related to non-alcoholic fatty liver disease, but not visceral adiposity index, in severely obese patients. Rev. Esp. Enferm. Dig..

[67] Ebrahimi R., Shanaki M., Mohassel Azadi S., Bahiraee A., Radmard A.R., Poustchi H., Emamgholipour S. (2019). Low level of adiponectin predicts the development of Nonalcoholic fatty liver disease: Is it irrespective to visceral adiposity index, visceral adipose tissue thickness and other obesity indices?. Arch. Physiol. Biochem..

[68] Eremić-Kojić N., Đerić M., Govorčin M.L., Balać D., Kresoja M., Kojić-Damjanov S. (2018). Assessment of hepatic steatosis algorithms in non-alcoholic fatty liver disease. Hippokratia.

[69] Fedchuk L., Nascimbeni F., Pais R., Charlotte F., Housset C., Ratziu V. (2014). Performance and limitations of steatosis biomarkers in patients with nonalcoholic fatty liver disease. Aliment. Pharmacol. Ther..

[70] Feng R.N., Du S.S., Wang C., Li Y.C., Liu L.Y., Guo F.C., Sun C.H. (2014). Lean-non-alcoholic fatty liver disease increases risk for metabolic disorders in a normal weight Chinese population. World J. Gastroenterol..

[71] Fu C.P., Ali H., Rachakonda V.P., Oczypok E.A., DeLany J.P., Kershaw E.E. (2019). The ZJU index is a powerful surrogate marker for NAFLD in severely obese North American women. PLoS ONE.

[72] Izadi A., Gargari B.P., Aliasghari F., Ebrahimi S. (2018). Adipokines and visceral adiposity index in relation to clinical findings of NAFLD patients. Prog. Nutr..

[73] Karamfilova V., Gateva A., Alexiev A., Zheleva N., Velikova T., Ivanova-Boyanova R., Ivanova R., Cherkezov N., Kamenov Z., Mateva L. (2019). The association between retinol-binding protein 4 and prediabetes in obese patients with nonalcoholic fatty liver disease. Arch. Physiol. Biochem..

[74] Keating S.E., Parker H.M., Hickman I.J., Gomersall S.R., Wallen M.P., Coombes J.S., Macdonald G.A., George J., Johnson N.A. (2017). NAFLD in clinical practice: Can simple blood and anthropometric markers be used to detect change in liver fat measured by (1) H-MRS?. Liver Int..

[75] Li L., You W., Ren W. (2017). The ZJU index is a powerful index for identifying NAFLD in the general Chinese population. Acta Diabetol..

[76] Li M., Shu W., Zunong J., Amaerjiang N., Xiao H., Li D., Vermund S.H., Hu Y. (2021). Predictors of non-alcoholic fatty liver disease in children. Pediatr. Res..

[77] Lin I.T., Lee M.Y., Wang C.W., Wu D.W., Chen S.C. (2021). Gender Differences in the Relationships among Metabolic Syndrome and Various Obesity-Related Indices with Nonalcoholic Fatty Liver Disease in a Taiwanese Population. Int. J. Environ. Res. Public Health.

[78] Musso G., Cassader M., De Michieli F., Rosina F., Orlandi F., Gambino R. (2012). Nonalcoholic steatohepatitis versus steatosis: Adipose tissue insulin resistance and dysfunctional response to fat ingestion predict liver injury and altered glucose and lipoprotein metabolism. Hepatology.

[79] Okamura T., Hashimoto Y., Hamaguchi M., Obora A., Kojima T., Fukui M. (2020). The visceral adiposity index is a predictor of incident nonalcoholic fatty liver disease: A population-based longitudinal study. Clin. Res. Hepatol. Gastroenterol..

[80] Sheng G., Lu S., Xie Q., Peng N., Kuang M., Zou Y. (2021). The usefulness of obesity and lipid-related indices to predict the presence of Non-alcoholic fatty liver disease. Lipids Health Dis..

[81] Villanueva-Ortega E., Garcés-Hernández M.J., Herrera-Rosas A., López-Alvarenga J.C., Laresgoiti-Servitje E., Escobedo G., Queipo G., Cuevas-Covarrubias S., Garibay-Nieto G.N. (2019). Gender-specific differences in clinical and metabolic variables associated with NAFLD in a Mexican pediatric population. Ann. Hepatol..

[82] Vural Keskinler M., Mutlu H.H., Sirin A., Erkalma Senates B., Colak Y., Tuncer I., Oguz A. (2021). Visceral Adiposity Index As a Practical Tool in Patients with Biopsy-Proven Nonalcoholic Fatty Liver Disease/Nonalcoholic Steatohepatitis. Metab. Syndr. Relat. Disord..

[83] Zaki M., Amin D., Mohamed R. (2020). Body composition, phenotype and central obesity indices in Egyptian women with non-alcoholic fatty liver disease. J. Complement Integr. Med..

[84] Eslam M., Sanyal A.J., George J., International Consensus P. (2020). MAFLD: A Consensus-Driven Proposed Nomenclature for Metabolic Associated Fatty Liver Disease. Gastroenterology.

[85] Eslam M., Newsome P.N., Sarin S.K., Anstee Q.M., Targher G., Romero-Gomez M., Zelber-Sagi S., Wai-Sun Wong V., Dufour J.F., Schattenberg J.M. (2020). A new definition for metabolic dysfunction-associated fatty liver disease: An international expert consensus statement. J. Hepatol..

[86] Kim D., Chung G.E., Kwak M.S., Seo H.B., Kang J.H., Kim W., Kim Y.J., Yoon J.H., Lee H.S., Kim C.Y. (2016). Body Fat Distribution and Risk of Incident and Regressed Nonalcoholic Fatty Liver Disease. Clin. Gastroenterol. Hepatol..

[87] Al-Daghri N.M., Al-Attas O.S., Alokail M., Alkharfy K., Wani K., Amer O.E., Ul Haq S., Rahman S., Alnaami A.M., Livadas S. (2014). Does visceral adiposity index signify early metabolic risk in children and adolescents?: Association with insulin resistance, adipokines, and subclinical inflammation. Pediatr. Res..

[88] Maffeis C., Manfredi R., Trombetta M., Sordelli S., Storti M., Benuzzi T., Bonadonna R.C. (2008). Insulin sensitivity is correlated with subcutaneous but not visceral body fat in overweight and obese prepubertal children. J. Clin. Endocrinol. Metab..

[89] Amato M.C., Giordano C. (2014). The current version of the visceral adiposity index is not suitable for application in pediatric populations: Comments on the article by Al-Daghri et al. Pediatr. Res..

[90] Hernández M.J.G., Huerta S.F. (2018). Pediatric Visceral Adiposity Index Adaptation Correlates with Homa-Ir, Matsuda, and Transaminases. Endocr. Pract..

[91] Jayakumar S., Harrison S.A., Loomba R. (2016). Noninvasive Markers of Fibrosis and Inflammation in Nonalcoholic Fatty Liver Disease. Curr. Hepatol. Rep..

[92] Vali Y., Lee J., Boursier J., Spijker R., Verheij J., Brosnan M.J., Anstee Q.M., Bossuyt P.M., Zafarmand M.H., on behalf of the LITMUS Systematic Review Team (2021). FibroTest for Evaluating Fibrosis in Non-Alcoholic Fatty Liver Disease Patients: A Systematic Review and Meta-Analysis. J. Clin. Med..

[93] Wong V.W., Adams L.A., de Ledinghen V., Wong G.L., Sookoian S. (2018). Noninvasive biomarkers in NAFLD and NASH—current progress and future promise. Nat. Rev. Gastroenterol. Hepatol..

[94] Ismaiel A., Leucuta D.C., Popa S.L., Fagoonee S., Pellicano R., Abenavoli L., Dumitrascu D.L. (2020). Noninvasive biomarkers in predicting non-alcoholic steatohepatitis and assessing liver fibrosis: Systematic review and meta-analysis. Panminerva. Med..

[95] Castera L., Friedrich-Rust M., Loomba R. (2019). Noninvasive Assessment of Liver Disease in Patients with Nonalcoholic Fatty Liver Disease. Gastroenterology.

