# Supplementary material for: The Visceral Adiposity Index in Non-Alcoholic Fatty Liver Disease and Liver Fibrosis—Systematic Review and Meta-Analysis
Source: Biomedicines. 2021 Dec 13;9(12):1890. doi: 10.3390/biomedicines9121890 (PMC8698356; doi:10.3390/biomedicines9121890)
Supplement: Supplementary file 1 [file biomedicines-09-01890-s001.zip › biomedicines-1476066-supplementary.pdf]

Search Strategy

PubMed:

(("visceral adiposity index") OR ("visceral adiposity index"[All Fields]) OR ("VAI")) AND (("Non-alcoholic Fatty Liver Disease"[Mesh]) OR ("Non-alcoholic Fatty Liver Disease"[All Fields]))

EMBASE:

('visceral adiposity index'/exp OR 'visceral adiposity index' OR 'VAI') AND ('Non-alcoholic Fatty Liver Disease'/exp OR 'Non-alcoholic Fatty Liver Disease')

Scopus (All fields):

(("visceral adiposity index") OR ("visceral adiposity index"[All Fields]) OR ("VAI")) AND (("Non-alcoholic Fatty Liver Disease"[Mesh]) OR ("Non-alcoholic Fatty Liver Disease"[All Fields]))

Cochrane Library:

Visceral adiposity index AND Non-alcoholic fatty liver disease

Supplementary Table S1. Visceral Adiposity Index in NAFLD

First Author / Year / Country	Study Design	Study Characteristics	Main Findings
**Musso et al. / 2012 / Italy (1)**	Observational study	• **Total Subjects:** 80<br>• **Population:** Patients referred for chronic liver enzyme elevation<br>• **NAFLD:** 40 (47.0%)<br>• **Mean age (years):** NASH: 47±4; SS: 47±4; Controls: 50±3<br>• **Gender (males):** 53.8.6 (30.5%)<br>• **BMI:** NASH: 25.2±1.6; SS: 25.1±1.5 Controls: 25.1±1.6<br>• **NAFLD diagnosis:** Histological<br>• **VAI - Mean ± SD / Median (IQR):** NASH: 1.19±0.34; SS: 1.16±0.28; Controls: 1.15±0.33<br>• **VAI – AUC:** -	NASH patients have a greater potential for developing T2DM and CVD than SS regardless of its association with obesity and metabolic syndrome.
**Petta et al. / 2011 / Italy (2)**	Cross-sectional	• **Total Subjects:** 142<br>• **Population:** Recruited patients with confirmed NAFLD via liver biopsy less than 6 months ago<br>• **NAFLD:** 100%<br>• **Mean age (years):** NAFLD: 45.4±13.0;<br>• **Gender (males):** 95 (66.9%)<br>• **BMI:** NAFLD: 16 (<25); 65 (25-29.9); 61 (>= 30)<br>• **NAFLD diagnosis:** Histological<br>• **VAI - Mean ± SD / Median (IQR):** 2.41±1.55<br>• **VAI – AUC:** 0.715	In NAFLD patients, visceral adiposity index is an expression of both qualitative and quantitative adipose tissue dysfunction and, together with insulin resistance, is independently correlated with significant fibrosis.
**Vongsuvanh et al. / 2012 / Australia (3)**	Cross-sectional	• **Total Subjects:** 319<br>• **Population:** Patients with biopsy-proven NAFLD recruited from tertiary clinic<br>• **NAFLD:** 190 (59.5%)<br>• **Mean age (years):** NAFLD: 49.4±13.0; SS: 48±11; Controls: 47±11<br>• **Gender (males):** NAFLD: 110 (57.8%); Controls: 71 (55.0%)<br>• **BMI:** NASH: 31.2±4.7; SS: 30.3±5.5; Controls: 26.9±4.8<br>• **NAFLD diagnosis:** Histological<br>• **VAI - Mean ± SD / Median (IQR):** Controls: 1.5±.1.1; SS: 2.3±1.6; NASH: 3.2±2.5<br>• **VAI – AUC:** 0.73 (distinguishing NASH vs non-NASH); 0.53 (severe vs non-severe fibrosis, non-significant p-value)	VAI is not associated with steatosis, inflammation or fibrosis in NAFLD. VAI is not powerful than WC in discriminating steatosis from steatohepatitis.
**Díez-Rodríguez et al. / 2014 / Spain (4)**	Cross-sectional	• **Total Subjects:** 139<br>• **Population:** Patients who underwent bariatric surgery<br>• **NAFLD:** Hepatic steatosis: 115 (89.9%); NASH: 25 (18.0%)<br>• **Mean age (years):** 43.81±10.6<br>• **Gender (males):** 39 (28.1%)<br>• **BMI:** 46.83±6<br>• **NAFLD diagnosis:** Histological<br>• **VAI - Mean ± SD / Median (IQR):** 2.36±1.36<br>• **VAI – AUC:** 0.71 (prediction of NASH)	VAI was associated with HOMA and metabolic syndrome but liver histology. HOMA, metabolic syndrome and WC were related to liver histology in patients with severe obesity.
**Feng et al. / 2014 / China (5)**	Observational study	• **Total Subjects:** 1779<br>• **Population:** Randomly selected patients who received physical examination at the second affiliated hospital of Harbin Medical university<br>• **NAFLD:** 898 (50.4%)	Normal weight individuals are more likely to have diabetes, hypertension and MetS if they have NAFLD. NAFLD is more dangerous in overweight-obese than lean NAFLD.

		• **Mean age (years):** Controls Lean-NAFLD: 43±11.59; Lean-NAFLD: 48.17±10.5; Controls Overweight-obese-NAFLD: 46.72±11.5; Overweight-obese-NAFLD: 46.92±11.19<br>• **Gender (males):** NAFLD: 624.9 (69.4%); Controls: 316 (35.8%)<br>• **BMI:** Controls Lean-NAFLD: 21.37±1.71; Lean-NAFLD: 22.74±1.13; Controls Overweight-obese-NAFLD: 25.98±1.66; Overweight-obese-NAFLD: 27.57±2.63<br>• **NAFLD diagnosis:** ultrasonography, alcohol consumption, absence of viral hepatitis, hepatolenticular degeneration, autoimmune diseases, total enteral nutrition or hepatotoxic drugs.<br>• **VAI - Mean ± SD / Median (IQR):** Controls Lean-NAFLD: 1.52±1.99; Lean-NAFLD: 2.04±2.21; Controls Overweight-obese-NAFLD: 1.85±2.32; Overweight-obese-NAFLD: 2.08±1.50<br>• **VAI – AUC:** -	
**Fedchuk et al. / 2014 / France (6)**	Retrospective study	• **Total Subjects:** 324<br>• **Population:** With liver biopsy between 2000 and 2010 for clinical and/or ultrasonographic suspicion of NAFLD<br>• **NAFLD:** 171 (53%)<br>• **Mean age (years):** 54 (45-60)<br>• **Gender (males):** 206 (64%)<br>• **BMI:** 29 (26-33)<br>• **NAFLD diagnosis:** Histology, clinical, ultrasonography<br>• **VAI - Mean ± SD / Median (IQR):** 1.8 (1.2-3.2)<br>• **VAI – AUC:** 0.92 (accuracy of steatosis presence); 0.59 (predicting steatosis > 33% and distinguishing between severe and non-severe)	All five (AUROC for FLI, LFS, HSI, VAI, TyG) biomarkers can diagnose steatosis and are correlated with insulin resistance. They are cofounded by fibrosis and inflammation, do not accurately quantify steatosis (this may limit their clinical utility).
**Ercin et al. / 2015 / Turkey (7)**	Observational study	• **Total Subjects:** 215<br>• **Population:** biopsy-proven NAFLD male patients<br>• **NAFLD:** NAFLD: 82.9 (38.6%); NASH: 50.9 (23.7%); SS: 81.0 (37.7%)]<br>• **Mean age (years):** 32.11 (20-53)<br>• **Gender (males):** 100%<br>• **BMI:** 28.31 (21.2-38.3)<br>• **NAFLD diagnosis:** Histology<br>• **VAI - Mean ± SD / Median (IQR):** 3.04 (0.24-12.01)<br>• **VAI – AUC:** -	VAI is not related to the severity of hepatic inflammation or fibrosis in nondiabetic patients with NAFLD. The lack of association between the adipocytokines and VAI also implies that the VAI may not be a significant indictor of the adipocyte functions.
**Keating et al. / 2017 / Australia (8)**	Observational study	• **Total Subjects:** 97<br>• **Population:** Adult man and women (18-60) with BMI > 25.0 kgm^2 recruited from noticeboards, electronic bulletins and clinical databases from 2011 to 2015<br>• **NAFLD:** 43 (44.3%)<br>• **Mean age (years):** NAFLD: 42.3±12.0; non-NAFLD: 37.7±10.8<br>• **Gender (males):** All: 63 (64.9%); NAFLD: 27 (62.7%); non-NAFLD: 36 (66.66%)<br>• **BMI:** NAFLD: 32.2±5.3; non-NAFLD: 29.1±3.1<br>• **NAFLD diagnosis:** Magnetic resonance imaging (MRI) and proton magnetic resonance spectroscopy (H-MRS); Steatosis biomarkers (NAFLD Liver Fat Score; LFE, FLI; HIS; VAI)<br>• **VAI - Mean ± SD / Median (IQR):** NAFLD: 2.4±1.8; non-NAFLD: 1.5±0.7<br>• **VAI – AUC:** -	Only HSI, FLI and WC were associated with changes in liver fat with a weak-moderate correlation. Only chance in WC significantly affected liver fat and WC-AUROC for the presence of steatosis.<br>no association between the change in VAI and change in liver fat.
**Li et al. / 2017 / China (9)**	Cross-sectional	• **Total Subjects:** 19804<br>• **Population:** Patients recruited from the First affiliated hospital Chongqing Medical University without a history of high alcohol intake, history of viral hepatitis, autoimmune hepatitis, drug-induced liver disease or any other chronic liver disease.<br>• **NAFLD:** 7324 (36.9%)<br>• **Mean age (years):** NAFLD: 46.75±14.86; non-NAFLD: 46.79±12.83<br>• **Gender (males):** Total: 15067 (76.0%); NAFLD: 5575 (75.5%)<br>• **BMI:** Control: 22.7±5.77; NAFLD: 26.10±2.73	ZJU index has a better predictive ability to distinguish NAFLD and diagnosis ability than VAI.

		• **NAFLD diagnosis:** Following the guidelines of the Chinese Liver Disease Association (biochemistry and liver ultrasonography)<br>• **VAI - Mean ± SD / Median (IQR):** 1.89 (Performance for identifying NAFLD)<br>• **VAI – AUC:** 0.747	
**Eremić-Kojić et al. / 2018 / Serbia (10)**	Cross-sectional	• **Total Subjects:** 77<br>• **Population:** Patients withs abdominal obesity (WC >= 102 cm for males and WC >= 88 cm for females)<br>• **NAFLD:** 44 (57.1%)<br>• **Mean age (years):** NAFLD: 34.38±9.73; non-NAFLD: 286.95±4.79<br>• **Gender (males):** 35 (45.4%)<br>• **BMI:** Control: 22.7±5.77; non-NAFLD: 26.10±2.73<br>• **NAFLD diagnosis:** Hepatic steatosis detection using ultrasonography using the National Health and Nutrition Examination Survey III<br>• **VAI - Mean ± SD / Median (IQR):** Control: 2.72±3.10; NAFLD: 2.87±2.32<br>• **VAI – AUC:** -	There is a positive statistically significant correlation between the degree of NAFLD determined by ultrasound and FLI, TyG, LAP, and HSI. There were no significant correlations between the degree of NAFLD and VAI.
**Izadi et al. / 2018 / Iran (11)**	Cross-sectional	• **Total Subjects:** 83<br>• **Population:** Patients from the University Hospital in Jahrom, between 20 and 50 years with confirmed documented diagnosis of NAFLD.<br>• **NAFLD:** 100%<br>• **Mean age (years):** NAFLD: 36.71±7.21<br>• **Gender (males):** 42 (50.6%)<br>• **BMI:** NAFLD: 29.41±4.18<br>• **NAFLD diagnosis:** Ultrasonography and Histology<br>• **VAI - Mean ± SD / Median (IQR):** NAFLD: 7.70±3.09 (Male) / 10.04±2.84 (Female)<br>• **VAI – AUC:** -	VAI is in positive significant association with the severity of liver fibrosis as well as AST activity in male patents.
**Xu et al. / 2018 / China (12)**	Prospective cohort study	• **Total Subjects:** 4809<br>• **Population:** individuals who participated in a medical checkup program conducted by the Beijing Xiaotangshan Hospital beginning on January 1, 2012<br>• **NAFLD:** 100%<br>• **Mean age (years):** Baseline: 48.69±15.19; Men: 50.86±15.95; Women: 45.24±13.31<br>• **Gender (males):** 2870 (59.68%)<br>• **BMI:** Quartile 1: 22.69±2.78; Quartile 2: 23.55±2.61; Quartile 3: 24.27±2.56; Quartile 4: 24.89±2.43<br>• **NAFLD diagnosis:** alcohol intake in questionnaire to diagnose the NAFLD and the Chinese criteria for ultrasound diagnosis of fatty liver were used in this study.<br>• **VAI - Mean ± SD / Median (IQR):** Quartile 1: 1.42 (1.24-1.64); Quartile 2: 1.73 (1.51-1.99); Quartile 3: 2.13 (1.51-1.99); Quartile 4: 2.13 (1.86-2.45)<br>• **VAI – AUC:** -<br>• **Follow up:** 4 years	The VAI level is an independent risk factor of NAFLD and that there is a dose-response relationship between VAI level and NAFLD risk.
**Ebrahimi et al. / 2019 / Iran (13)**	Cross-sectional	• **Total Subjects:** 124<br>• **Population:** Recruited patients recently diagnosed and were recruited from the outpatient clinics of Shariati Hospital.<br>• **NAFLD:** 82 (66.1%); NAFLD-only: 41 (33%) ; NAFLD+T2D: 41 (33%)<br>• **Mean age (years):** NAFLD: 51 (48–54.5); NAFLD+T2D: 53 (46.5–58); Healthy: 51 (48–60.75)<br>• **Gender (males):** 100%<br>• **BMI:** NAFLD: 28.94 ± 3.15**;** NAFLD+T2D: 29.24 ± 3.85; Healthy: 24.88±3.71<br>• **NAFLD diagnosis:** Biochemistry; Ultrasonography<br>• **VAI - Mean ± SD / Median (IQR):** NAFLD: 4.243±1.43; NAFLD+T2D: 5.13±3.04; Healthy: 3.247±1.35;<br>• **VAI – AUC:** -	VAI is the best predictor of adiponectin level in the whole population and also in the combination of patients' groups.<br>Lower level of adiponectin increased the risk of developing NAFLD, T2DM, and NAFLD-T2D independently to obesity indices or IR

**Fu et al. / 2019 / USA (14)**	Retrospective analysis of a prospectively-collected cohort	• **Total Subjects:** 107<br>• **Population:** women between the age of 30 and 55 with obesity class II (BMI 35– 39.9 kg/m2) and class III (BMI ≥ 40 kg/m2) recruited at University of Pittsburgh Medical Center (RENEW clinical trial)<br>• **NAFLD:** 40 (37.8%)<br>• **Mean age (years):** NAFLD: 46±6; non-NAFLD: 47±7<br>• **Gender (males):** 0%<br>• **BMI:** NAFLD: 46.0±5.7; non-NAFLD: 42.1±4.8<br>• **NAFLD diagnosis:** Hepatic steatosis measured by Liver/Spleen attenuation ratio from unenhanced abdominal computed tomography<br>• **VAI - Mean ± SD / Median (IQR):** NAFLD: 1.7±1.0; non-NAFLD: 1.5±1.2<br>• **VAI – AUC:** 0.621	We found that the ZJU index, HSI, and LAPI were significantly higher in the NAFLD group, but not VAI.
**Karamfilova et al. / 2019 / Bulgaria (15)**	Observational study	• **Total Subjects:** 79<br>• **Population:** Caucasian subjects aged between 25 to 65 years old with NAFLD recruited in Endocrinology.Clinic at the University Hospital Alexandrovska<br>• **NAFLD:** 100%<br>• **Mean age (years):** 50.95 ± 11.11; NAFLD: 51.20±12.97; NAFLD-prediabetes: 50.68±8.83<br>• **Gender (males):** 6 (7.5%)<br>• **BMI:** NAFLD: 36.6±6; NAFLD-prediabetes: 36.33±5.24<br>• **NAFLD diagnosis:** Ultrasonography<br>• **VAI - Mean ± SD / Median (IQR):** NAFLD: 1.71±0.9; NAFLD-prediabetes: 3.04±1.49<br>• **VAI – AUC: -**	Significantly higher levels of VAI, very low-density lipoproteins (VLDL), triglycerides (TG), blood glucose, insulin from OGTT, HOMA-IR, as well as lower levels of high density lipo- proteins (HDL), and Quicki index were found in obese NAFLD patients with prediabetes (group 2) than group 1 patients
**Villanueva-Ortega et al. / 2019 / Mexico (16)**	Cross-sectional comparative study	• **Total Subjects:** 194<br>• **Population:** Eutrophic, overweight, and individuals with obesity and ranging in age from 6–18 years were consecutively included from January 2015 to September 2016. All patients included in this study were born and raised in Mexico City and the suburban area.<br>• **NAFLD:** 36 (18.5%)<br>• **Mean age (years):** NAFLD male: 11.08±2.04; non-NAFLD male: 10.26±2.47; NAFLD female: 10.83±3.27; non-NAFLD female: 10.85±2.64<br>• **Gender (males):** 102 (52.5%)<br>• **BMI: (**percentiles) NAFLD male: 97.33±2.19; non-NAFLD male: 85.85±22.4; NAFLD female: 97.82±1.56; non-NAFLD female: 81.72±23.75<br>• **NAFLD diagnosis:** Ultrasonography<br>• **VAI - Mean ± SD / Median (IQR):** NAFLD male: 2.87±1.97; non-NAFLD male: 2.57±1.89; NAFLD female: 4.30±1.75; non-NAFLD female: 2.38±1.66<br>• **VAI – AUC:** NAFLD-female: 2.33	NAFLD is more frequent in boys, only ALT, and no other clinical or metabolic variables, were associated with NAFLD in these patients. HOMA-IR, VAI, triglycerides levels, and ALT were associated with NAFLD only in girls.
**Cen et al. / 2020 / China (17)**	Retrospective cross-sectional study	• **Total Subjects:** 16,468<br>• **Population:** Adults (18–75 years old) who presented for their annual health examinations at the First Affiliated Hospital of Zhejiang University School of Medicine in 2014 and 2016. The data were extracted retrospectively from the health examination database.<br>• **NAFLD:** 6261 (38.0%)<br>• **Mean age (years):** Overall: 45.64 ± 11.02; NAFLD: 47.89±10.27; non-NAFLD: 44.25±11.24<br>• **Gender (males):** 9452 (57.3%)<br>• **BMI: Overall:** 23.90±3.29; NAFLD: 26.11±2.96; non-NAFLD: 22.54±2.68<br>• **NAFLD diagnosis:** Ultrasonography, staged according to the Chinese Liver Disease Association<br>• **VAI - Mean ± SD / Median (IQR):**<br>• **VAI – AUC:** 0.752 (NAFLD prediction)	Study developed a simple CLN for predicting NAFLD in a large Chinese population. The AUROC of the CLN was better than the FLI, the VAI, the HSI and the TyG index.

**Coccia et al. / 2020 / Italy (18)**	Observational study	• **Total Subjects:** 90<br>• **Population:** morbid obese patients undergoing Bariatric Surgery (BS) in Latin, in Department of Medico-Surgical Sciences and Biotechnologies Division of General Surgery and Bariatric Center of Excellence, "La Sapienza".<br>• **NAFLD:** 69,3 (77%); NFL: 47,7 (53%); NASH: 21,6 (24%)<br>• **Mean age (years):** Entire cohort: 40.29±11.65; Control: 41.11±12.52; NAFLD: 39.07±11.28; NASH: 42.47±11.96<br>• **Gender (males):** Entire cohort: 73 (81.1%); Control: 19 (90.4%); NAFL: 35 (74.4%); NASH: 19 (86.3%)<br>• **BMI:** Entire cohort: 43,18±5.92; Control: 41.82±5.30; NAFL: 44.02±5.94; NASH: 42.43±6.36<br>• **NAFLD diagnosis:** Histological<br>• **VAI - Mean ± SD / Median (IQR):** Entire cohort: 5.55±2.92; Control: 4.38±2.93; NAFL: 5.52±2.77; NASH: 7.54±2.46<br>• **VAI – AUC:** 0.72	All steatosis scores assessed were significantly higher in NAFLD patients than controls but not significantly different between NAFL and NASH, except VAI, that increased significantly in NASH than NAFL.
**Okamura et al. / 2020 /Japan (19)**	Historical cohort study	• **Total Subjects:** 8399<br>• **Population:** Data of individuals who participated in the medical examination program, a survey was carried on the impact of VAI on the risk of incident NAFLD, using the (NAFLD in the Gifu Area, Longitudinal Analysis) database.<br>• **NAFLD:** 1078 (12.8%); Men: 737 (68.3%); Women: 341 (31.6%)<br>• **Mean age (years):** Men: 41.7±9.0; Women: 41.5±8.8<br>• **Gender (males):** 3773 (44.9%)<br>• **BMI:** Men: 22.1±2.4; Women: 20.6±2.6<br>• **NAFLD diagnosis:** Ultrasonography<br>• **VAI - Mean ± SD / Median (IQR):** Men: 0.69 (0.43-1.13); Women: 0.54 (0.36-0.83)<br>• **VAI – AUC: -**<br>• **Follow up:** median follow-up for: Men: 4.5-year; Women: 4.9-year	VAI can be a predictor of incident NAFLD.
**Zaki et al. / 2020 / Egypt (20)**	Observational study	• **Total Subjects:** 200<br>• **Population:** Cases of premenopausal women with NAFLD have been collected from different clinics of the National Research Centre, Giza, between September 2018 and December 2019<br>• **NAFLD:** 100 (50%)<br>• **Mean age (years): - (**matched with healthy controls)<br>• **Gender (males):** 0%<br>• **BMI:** NAFLD: 32.8±5.56; non-NAFLD: 24.7±3.51<br>• **NAFLD diagnosis:** hepatomegaly, elevated hepatic AST and ALT, fatty liver changes and ultrasonography<br>• **VAI - Mean ± SD / Median (IQR):** NAFLD: 4.9±1.90; non-NAFLD: 1.30±0.68<br>• **VAI – AUC:** 0.920	VAI is the most reliable parameter for predicting NAFLD followed by WHR and WHtR.
**Li / 2021 / China (21)**	Population cohort study	• **Total Subjects:** 1350<br>• **Population:** Children aged 6–8 years from the child cohort designed to study sensitization, puberty, obesity and cardiovascular risk (PROC) in the urban area of Shunyi District, Beijing.<br>• **NAFLD:** 95 (7.0%); Male: 70 (10.4%); Female: 25 (3.7%)<br>• **Mean age (years):** Normal: 6.66±0.30; NAFLD: 6.70±0.30<br>• **Gender (males):** 674 (49.9%); Normal: 604 (48.13%); NAFLD: 70 (73.6%)<br>• **BMI:** -<br>• **NAFLD diagnosis:** Ultrasonography<br>• **VAI - Mean ± SD / Median (IQR):** Normal: 0.57±0.36; NAFLD: 0.85±0.60;<br>• **VAI – AUC:** Fitted VAI-AUC for Males: 0.892 (0.845–0.938); Females: 0.928 (0.864–0.992)	Fitted and empirical curves showed that the predictive ability of VAI is better among girls than boys.<br>VAI was not the best indicator for predicting NAFLD.
**Lin / 2021 / China (22)**	Observational study	• **Total Subjects:** 1969<br>• **Population:** subjects who took part a health survey from June 2016 to September 2018 in southern Taiwan and were willing to participate in the study<br>• **NAFLD:** 826 (42.0%)<br>• **Mean age (years):** 54.9±13.5 years; non-NAFLD male: 56.36 ± 14.47; NAFLD male: 53.86±13.41; non-NAFLD female: 53.20±13.85; NAFLD female: 57.03±11.77<br>• **Gender (males):** Total: 764 (38.8%); non-NAFLD: 410 (53.6%); NAFLD: 354 (46.3%)	MetS, high BMI, high WHtR, high WHR, high LAP, high BRI, high CI, high VAI, high BAI, high AVI, high TyG index, and high HSI were significantly associated with NAFLD. Those values increased with the severity of NAFLD.

		• **BMI:** non-NAFLD male: 24.42±3.25; NAFLD male: 26.97±3.38; non-NAFLD female: 23.11±3.45; NAFLD female: 26.64±3.91<br>• **NAFLD diagnosis:** Ultrasonography<br>• **VAI - Mean ± SD / Median (IQR):** non-NAFLD male: 3.41±2.66; NAFLD male: 5.84±5.11; non-NAFLD female: 3.17±2.43; NAFLD female: 6.16±8.21<br>• **VAI – AUC:** Male: 0.715; Female: 0.732	
**Sheng et al. / 2021 / China (23)**	Observational study	• **Total Subjects:** 14,281<br>• **Population:** Subjects of this study were from the NAGALA study.<br>• **NAFLD:** 2507 (17.5%); Males: 2029 (14.2%); Females: 478 (3.35%)<br>• **Mean age (years):** non-NAFLD male: 43.71±9.27; NAFLD male: 44.11±8.20; non-NAFLD female: 42.89±8.72; NAFLD female: 47.64±8.29<br>• **Gender (males): 7411;** NAFLD**: 2029**<br>• **BMI:** non-NAFLD male: 22.12±2.42; NAFLD male: 25.48±3.02; non-NAFLD female: 20.67±2.57; NAFLD female: 25.58±3.57<br>• **NAFLD diagnosis:** Ultrasonography<br>• **VAI - Mean ± SD / Median (IQR):** non-NAFLD male: 0.74 (0.47-1.21); NAFLD male: 1.49 (0.95-2.31); non-NAFLD female: 0.57 (0.39-0.88); NAFLD female: 1.37 (0.89-2.05)<br>• **VAI – AUC:** 0.8000; Male: 0.7565; Female: 0.8281	TyG index-related parameters may be the best choice for NAFLD risk screening.<br>VAI was weakly correlated with NAFLD in the whole population.<br>People with NAFLD had a higher VAI.
**Vural Keskinler et al. / 2021 / Turkey (24)**	Cross-sectional	• **Total Subjects:** 114<br>• **Population:** Study included individuals from the gastroenterology service with high transaminases for minimum 6 months, no hepatotoxic drug or disease that could cause NAFLD and diagnosed with liver biopsy.<br>• **NAFLD:** 57 (50%); NASH: 47 (84.5%); SS: 10 (15.5%);<br>• **Mean age (years):** Controls: 41.35±11.91; NASH: 43.79±9.35; SS: 42.4±12.12; NAFLD: 43.54±9.78<br>• **Gender (males):** Controls: 28 (24.5%); NAFLD: 56 (49.1%)<br>• **BMI:** Controls: 24.21±4.52; NASH: 32.17±5.56; SS: 29.2±4.2; NAFLD: 32.17±5.56<br>• **NAFLD diagnosis:** Histological<br>• **VAI - Mean ± SD / Median (IQR):** Controls: 1.60±0.98; NASH: 3.60±3.35; SS: 3.00±1.42; NAFLD: 3.5±3.09<br>• **VAI – AUC:** 1.78	VAI was increased in the NAFLD cases, especially in NASH cases.<br>No significant relationship was found between the histological parameters and VAI scores.
ALT - alanine aminotransferase, AST - aspartate aminotransferase, AUC - area under the curve, AUROC - Area Under the Receiver Operating Characteristic curve, AVI - Abdominal volume index, BMI - body-mass index, CI - conicity interval, CLN - clinical and laboratory-based nomogram, CVD - cardiovascular disease, FLI - fatty liver index, HOMA - homeostatic model assessment, HSI - hepatic steatosis index, IQR - interquartile range, IR - Insulin resistance, LAP - Lipid accumulation product, LFS - Liver fat score, MetS - metabolic syndrome, NAFL - nonalcoholic fatty liver, NAFLD - nonalcoholic fatty liver disease, NASH - nonalcoholic steatohepatitis, OGTT - oral glucose tolerance test, SD - standard deviation, SS - simple steatosis, T2DM - type 2 diabetes mellitus, TyG - triglycerides, VAI - Visceral adiposity index, WC - waist circumference, WHR - waist-to-hip ratio, WHtR - waist-to-height ratio, ZJU index - Zhejiang University index.			

Supplementary Table S2. QUADAS-2 tool for evaluating the methodological quality of included studies

**Criteria**	*Musso 2012 (25)*	*Petta 2012 (26)*	*Vongsuvanh 2012 (27)*	*Díez-Rodríguez 2014 (28)*	*Fedchuk 2014 (6)*	*Feng 2014 (29)*	*Ercin 2015 (30)*	*Keating 2017 (31)*	*Li 2017 (32)*
**Domain 1 – Patient selection**									
**A. Risk of bias**									
Was a consecutive or random sample of patients enrolled?	?	Yes	?	?	Yes	Yes	?	?	Yes
Was a case-control design avoided?	No	Yes	No	Yes	Yes	No	Yes	No	No
Did the study avoid inappropriate exclusions?	Yes	Yes	Yes	Yes	Yes	Yes	Yes	Yes	Yes
**Risk of bias assessment**	☹	☺	☹	☺	☺	☺	☺	☹	☺
**B. Applicability**	☺	☺	☺	☹	Low	☹	Low	☹	☹
**Domain 2 – Index test**									
**A. Risk of bias**									
Were the index test results interpreted without knowledge of the results of the reference standard?	Yes	Yes	Yes	Yes	Yes	Yes	Yes	Yes	Yes
If a threshold was used, was it pre-specified?	NA	NA	NA	NA	NA	NA	NA	NA	NA
**Risk of bias assessment**	☺	☺	☺	☺	☺	☺	☺	☺	☺
**B. Applicability**	☺	☺	☺	☺	☺	☺	☺	☺	☺
**Domain 3 – Reference standard**									
**A. Risk of bias**									
Is the reference standard likely to correctly classify the target condition?	Yes	Yes	Yes	Yes	Yes	No	Yes	Yes	No
Were the reference standard results interpreted without knowledge of the results of the index test?	Yes	Yes	Yes	Yes	Yes	Yes	Yes	Yes	Yes
**Risk of bias assessment**	☺	☺	☺	☺	☺	☹	☺	☺	☹
**B. Applicability**	☺	☺	☺	☺	☺	☺	☺	☺	☺
**Domain 4 – Flow and timing**									
**A. Risk of bias**									
Was there an appropriate interval between index test(s) and reference standard?	?	?	?	?	Yes	?	?	?	?
Did all patients receive a reference standard?	No	Yes	No	Yes	Yes	Yes	Yes	Yes	Yes
Did patients receive the same reference standard?	No	Yes	No	Yes	Yes	Yes	Yes	Yes	Yes
Were all patients included in the analysis?	Yes	Yes	Yes	Yes	Yes	Yes	Yes	Yes	Yes
**Risk of bias assessment**	☺	☺	☺	☺	☺	☺	☺	☺	☺

NA, not applicable; ☺, low risk; ☹, high risk; ?, unclear

Supplementary Table S2 (Cont'd). QUADAS-2 tool for evaluating the methodological quality of included studies

Criteria	*Eremić-Kojić 2018 (10)*	*Izadi 2018 (11)*	*Xu 2018 (33)*	*Ebrahimi 2019 (13)*	*Fu 2019 (34)*	*Karamfilova 2019 (15)*	*Villanueva-Ortega 2019 (35)*	*Cen 2020 (36)*	*Coccia 2020 (18)*
**Domain 1 – Patient selection**									
**C. Risk of bias**									
Was a consecutive or random sample of patients enrolled?	No	?	?	?	Yes	?	Yes	?	?
Was a case-control design avoided?	No	Yes	Yes	No	No	Yes	No	No	No
Did the study avoid inappropriate exclusions?	Yes	Yes	Yes	Yes	Yes	Yes	Yes	Yes	Yes
**Risk of bias assessment**	☹	☺	☺	☹	☺	☺	☺	☹	☹
**D. Applicability**	?	?	☹	?	☹	☺	☹	☹	☹
**Domain 2 – Index test**									
**C. Risk of bias**									
Were the index test results interpreted without knowledge of the results of the reference standard?	Yes	Yes	Yes	Yes	Yes	Yes	Yes	Yes	Yes
If a threshold was used, was it pre-specified?	NA	NA	NA	NA	NA	NA	NA	NA	NA
**Risk of bias assessment**	☺	☺	☺	☺	☺	☺	☺	☺	☺
**D. Applicability**	☺	☺	☺	☺	☺	☺	☺	☺	☺
**Domain 3 – Reference standard**									
**C. Risk of bias**									
Is the reference standard likely to correctly classify the target condition?	No	No	No	No	No	No	No	No	Yes
Were the reference standard results interpreted without knowledge of the results of the index test?	Yes	Yes	Yes	Yes	Yes	Yes	Yes	Yes	Yes
**Risk of bias assessment**	☹	☹	☹	☹	☹	☹	☹	☹	☺
**D. Applicability**	☺	☺	☺	☺	☺	☺	☺	☺	☺
**Domain 4 – Flow and timing**									
**B. Risk of bias**									
Was there an appropriate interval between index test(s) and reference standard?	Yes	?	?	?	?	?	?	?	?
Did all patients receive a reference standard?	Yes	Yes	Yes	Yes	Yes	Yes	Yes	Yes	Yes
Did patients receive the same reference standard?	Yes	Yes	Yes	Yes	Yes	Yes	Yes	Yes	Yes
Were all patients included in the analysis?	Yes	Yes	Yes	Yes	Yes	Yes	Yes	Yes	Yes
**Risk of bias assessment**	☺	☺	☺	☺	☺	☺	☺	☺	☺

NA, not applicable; ☺, low risk; ☹, high risk; ?, unclear

Supplementary Table S2 (Cont'd). QUADAS-2 tool for evaluating the methodological quality of included studies

**Criteria**	*Okamura 2020 (37)*	*Zaki 2020 (38)*	*Li 2021 (39)*	*Lin 2021 (40)*	*Sheng 2021 (41)*	*Vural Keskinler 2021 (24)*
**Domain 1 – Patient selection**						
**E. RoB**						
Was a consecutive or random sample of patients enrolled?	?	?	Yes	?	?	?
Was a case-control design avoided?	Yes	No	No	No	No	No
Did the study avoid inappropriate exclusions?	Yes	Yes	Yes	Yes	Yes	Yes
**Risk of bias assessment**	☺	☹	☺	☹	☹	☹
**F. Applicability**	☹	☺	☹	☹	☹	☺
**Domain 2 – Index test**						
**E. RoB**						
Were the index test results interpreted without knowledge of the results of the reference standard?	Yes	Yes	Yes	Yes	Yes	Yes
If a threshold was used, was it pre-specified?	NA	NA	NA	NA	NA	NA
**Risk of bias assessment**	☺	☺	☺	☺	☺	☺
**F. Applicability**	☺	☺	☺	☺	☺	☺
**Domain 3 – Reference standard**						
**E. RoB**						
Is the reference standard likely to correctly classify the target condition?	No	No	No	No	No	Yes
Were the reference standard results interpreted without knowledge of the results of the index test?	Yes	Yes	Yes	Yes	Yes	Yes
**Risk of bias assessment**	☹	☹	☹	☹	☹	☺
**F. Applicability**	☺	☺	☺	☺	☺	☺
**Domain 4 – Flow and timing**						
**C. RoB**						
Was there an appropriate interval between index test(s) and reference standard?	?	?	?	?	?	?
Did all patients receive a reference standard?	Yes	Yes	Yes	Yes	Yes	No
Did patients receive the same reference standard?	Yes	Yes	Yes	Yes	Yes	No
Were all patients included in the analysis?	Yes	Yes	Yes	Yes	Yes	Yes
**Risk of bias assessment**	☺	☺	☺	☺	☺	☺

NA, not applicable; ☺, low risk; ☹, high risk; ?, unclear

Supplementary Table S3. Meta-regression models adjusting for publication year, patient selection quality, standard quality and a multivariate model including the publication year and patient selection bias risk, for studies in adults

Characteristic MD (95% CI)	Publication Year	RoB in Patient Selection	RoB in Standard Quality	Multivariate
**Publication year**	0.167 (-0.027 - 0.360)			0.158 (-0.032 - 0.348)
**p-value**	0.083			0.093
**RoB in standard quality high vs. low/medium**		-0.938 (-2.522 - 0.645)		0.846 (-2.286 -0.594)
**p-value**		0.216		0.217
**RoB in patient selection high vs. low**			-0.224 (-1.747 - 1.298)	
**p-value**			0.750	
**AICc**	38.599	40.045	41.459	43.158
**H^2**	33.211	63.904	58.482	21.803
**I^2**	96.989	98.435	98.290	95.413
**p value cochran qe**	0.000	0.000	0.000	0.000
**p value cochran qm**	0.083	0.216	0.750	0.108
**tau^2**	0.943	1.096	1.273	0.868
**tau^2 se**	0.471	0.539	0.618	0.463

AIC – Akaike's Information Criteria**; CI –** Confidence interval**; MD –** Mean difference; **RoB** – Risk of bias; **se –** Standard error**.**

Supplementary Table S4. Meta-regression models adjusting for publication year, patient selection quality, standard quality and a multivariate model including the publication year and patient selection bias risk, for studies in adults and children

Characteristic MD (95% CI)	Publication Year	RoB in Patient Selection	RoB in Standard Quality	Multivariate
**Publication year**	0.129 (-0.054 - 0.313)			0.134 (-0.032 - 0.301)
**p-value**	0.150			0.104
**RoB in standard quality low/medium vs. high**		-0.993 (-2.247 - 0.261)		-1.032 (-2.199 -0.134)
**p-value**		0.110		0.077
**RoB in patient selection high vs. low**			-0.056 (-1.406 - 1.293)	
**p-value**			0.929	
**AICc**	43.476	42.964	45.475	44.140
**H^2**	66.067	57.870	87.086	30.348
**I^2**	98.486	98.272	98.852	96.705
**p value cochran qe**	0.000	0.000	0.000	0.000
**P value cochran qm**	0.150	0.110	0.929	0.073
**tau^2**	0.967	0.910	1.147	0.770
**tau^2 se**	0.437	0.412	0.509	0.372

AIC – Akaike's Information Criteria**; CI –** Confidence interval**; MD –** Mean difference; **RoB** – Risk of bias; **se –** Standard error**.**

References

1. Musso G, Cassader M, De Michieli F, Rosina F, Orlandi F, Gambino R. Nonalcoholic steatohepatitis versus steatosis: adipose tissue insulin resistance and dysfunctional response to fat ingestion predict liver injury and altered glucose and lipoprotein metabolism. Hepatology. 2012;56(3):933-42. doi: 10.1002/hep.25739.
2. Petta S, Amato M, Di Marco V, Cammà C, Pizzolanti G, Rosa Barcellona M, et al. Visceral adiposity index is associated with significant fibrosis in patients with nonalcoholic fatty liver disease. Hepatology. 2011;54:1131A. doi: 10.1002/hep.24666.
3. Vongsuvanh R, George J, McLeod D, van der Poorten D. Visceral adiposity index is not a predictor of liver histology in patients with non-alcoholic fatty liver disease. J Hepatol. 2012;57(2):392-8. doi: 10.1016/j.jhep.2012.03.013.
4. Díez-Rodríguez R, Ballesteros-Pomar MD, Calleja-Fernández A, González-De-Francisco T, González-Herráez L, Calleja-Antolín S, et al. Insulin resistance and metabolic syndrome are related to non-alcoholic fatty liver disease, but not visceral adiposity index, in severely obese patients. Rev Esp Enferm Dig. 2014;106(8):522-8.
5. Feng RN, Du SS, Wang C, Li YC, Liu LY, Guo FC, et al. Lean-non-alcoholic fatty liver disease increases risk for metabolic disorders in a normal weight Chinese population. World J Gastroenterol. 2014;20(47):17932-40. doi: 10.3748/wjg.v20.i47.17932.
6. Fedchuk L, Nascimbeni F, Pais R, Charlotte F, Housset C, Ratziu V. Performance and limitations of steatosis biomarkers in patients with nonalcoholic fatty liver disease. Aliment Pharmacol Ther. 2014;40(10):1209-22. doi: 10.1111/apt.12963.
7. Ercin CN, Dogru T, Genc H, Celebi G, Aslan F, Gurel H, et al. Insulin Resistance but Not Visceral Adiposity Index Is Associated with Liver Fibrosis in Nondiabetic Subjects with Nonalcoholic Fatty Liver Disease. Metab Syndr Relat Disord. 2015;13(7):319-25. doi: 10.1089/met.2015.0018.
8. Keating SE, Parker HM, Hickman IJ, Gomersall SR, Wallen MP, Coombes JS, et al. NAFLD in clinical practice: Can simple blood and anthropometric markers be used to detect change in liver fat measured by (1) H-MRS? Liver Int. 2017;37(12):1907-15. doi: 10.1111/liv.13488.
9. Li L, You W, Ren W. The ZJU index is a powerful index for identifying NAFLD in the general Chinese population. Acta Diabetol. 2017;54(10):905-11. doi: 10.1007/s00592-017-1024-8.
10. Eremić-Kojić N, Đerić M, Govorčin ML, Balać D, Kresoja M, Kojić-Damjanov S. Assessment of hepatic steatosis algorithms in non-alcoholic fatty liver disease. Hippokratia. 2018;22(1):10-6.
11. Izadi A, Gargari BP, Aliasghari F, Ebrahimi S. Adipokines and visceral adiposity index in relation to clinical findings of NAFLD patients. Progress in Nutrition. 2018;20:145-52. doi: 10.23751/pn.v20i2-S.6019.
12. Xu C, Ma Z, Wang Y, Liu X, Tao L, Zheng D, et al. Visceral adiposity index as a predictor of NAFLD: A prospective study with 4-year follow-up. Liver Int. 2018;38(12):2294-300. doi: 10.1111/liv.13941.
13. Ebrahimi R, Shanaki M, Mohassel Azadi S, Bahiraee A, Radmard AR, Poustchi H, et al. Low level of adiponectin predicts the development of Nonalcoholic fatty liver disease: Is it irrespective to visceral adiposity index, visceral adipose tissue thickness and other obesity indices? Arch Physiol Biochem. 2019:1-8. doi: 10.1080/13813455.2019.1661496.

14. Fu CP, Ali H, Rachakonda VP, Oczypok EA, DeLany JP, Kershaw EE. The ZJU index is a powerful surrogate marker for NAFLD in severely obese North American women. PLoS One. 2019;14(11):e0224942. doi: 10.1024/0300-9831/a000442 10.1371/journal.pone.0224942.
15. Karamfilova V, Gateva A, Alexiev A, Zheleva N, Velikova T, Ivanova-Boyanova R, et al. The association between retinol-binding protein 4 and prediabetes in obese patients with nonalcoholic fatty liver disease. Arch Physiol Biochem. 2019:1-6. doi: 10.1080/13813455.2019.1673429.
16. Villanueva-Ortega E, Garcés-Hernández MJ, Herrera-Rosas A, López-Alvarenga JC, Laresgoiti-Servitje E, Escobedo G, et al. Gender-specific differences in clinical and metabolic variables associated with NAFLD in a Mexican pediatric population. Ann Hepatol. 2019;18(5):693-700. doi: 10.1371/journal.pone.0224942
10.1016/j.aohep.2019.04.012.
17. Cen C, Wang W, Yu S, Tang X, Liu J, Liu Y, et al. Development and validation of a clinical and laboratory-based nomogram to predict nonalcoholic fatty liver disease. Hepatol Int. 2020;14(5):808-16. doi: 10.1007/s12072-020-10065-7.
18. Coccia F, Testa M, Guarisco G, Bonci E, Di Cristofano C, Silecchia G, et al. Noninvasive assessment of hepatic steatosis and fibrosis in patients with severe obesity. Endocrine. 2020;67(3):569-78. doi: 10.1007/s12020-019-02155-w.
19. Okamura T, Hashimoto Y, Hamaguchi M, Obora A, Kojima T, Fukui M. The visceral adiposity index is a predictor of incident nonalcoholic fatty liver disease: A population-based longitudinal study. Clin Res Hepatol Gastroenterol. 2020;44(3):375-83. doi: 10.1016/j.clinre.2019.04.002.
20. Zaki M, Amin D, Mohamed R. Body composition, phenotype and central obesity indices in Egyptian women with non-alcoholic fatty liver disease. J Complement Integr Med. 2020;18(2):385-90. doi: 10.1515/jcim-2020-0073.
21. Li M, Shu W, Zunong J, Amaerjiang N, Xiao H, Li D, et al. Predictors of non-alcoholic fatty liver disease in children. Pediatr Res. 2021. doi: 10.1038/s41390-021-01754-6.
22. Lin IT, Lee MY, Wang CW, Wu DW, Chen SC. Gender Differences in the Relationships among Metabolic Syndrome and Various Obesity-Related Indices with Nonalcoholic Fatty Liver Disease in a Taiwanese Population. Int J Environ Res Public Health. 2021;18(3). doi: 10.3390/ijerph18030857.
23. Sheng G, Lu S, Xie Q, Peng N, Kuang M, Zou Y. The usefulness of obesity and lipid-related indices to predict the presence of Non-alcoholic fatty liver disease. Lipids Health Dis. 2021;20(1):134. doi: 10.1186/s12944-021-01561-2.
24. Vural Keskinler M, Mutlu HH, Sirin A, Erkalma Senates B, Colak Y, Tuncer I, et al. Visceral Adiposity Index As a Practical Tool in Patients with Biopsy-Proven Nonalcoholic Fatty Liver Disease/Nonalcoholic Steatohepatitis. Metab Syndr Relat Disord. 2021;19(1):26-31. doi: 10.1089/met.2020.0054.
25. Musso G, Cassader M, De Michieli F, Rosina F, Orlandi F, Gambino R. Nonalcoholic steatohepatitis versus steatosis: Adipose tissue insulin resistance and dysfunctional response to fat ingestion predict liver injury and altered glucose and lipoprotein metabolism. Hepatology. 2012;56(3):933-42. doi: 10.1002/hep.25739.

26. Petta S, Amato MC, Di Marco V, Cammà C, Pizzolanti G, Barcellona MR, et al. Visceral adiposity index is associated with significant fibrosis in patients with non-alcoholic fatty liver disease. Aliment Pharmacol Ther. 2012;35(2):238-47. doi: 10.1111/j.1365-2036.2011.04929.x.
27. Vongsuvanh R, George J, McLeod D, Van Der Poorten D. Visceral adiposity index is not a predictor of liver histology in patients with non-alcoholic fatty liver disease. J Hepatol. 2012;57(2):392-8. doi: 10.1016/j.jhep.2012.03.013.
28. Díez-Rodríguez R, Ballesteros-Pomar MD, Calleja-Fernández A, González-De-Francisco T, González-Herráez L, Calleja-Antolín S, et al. Insulin resistance and metabolic syndrome are related to non-alcoholic fatty liver disease, but not visceral adiposity index, in severely obese patients. Rev Esp Enferm Dig. 2014;106(8):522-8.
29. Feng RN, Du SS, Wang C, Li YC, Liu LY, Guo FC, et al. Lean-non-alcoholic fatty liver disease increases risk for metabolic disorders in a normal weight Chinese population. World J Gastroenterol. 2014;20(47):17932-40. doi: 10.3748/wjg.v20.i47.17932.
30. Ercin CN, Dogru T, Genc H, Celebi G, Aslan F, Gurel H, et al. Insulin resistance but not visceral adiposity index is associated with liver fibrosis in nondiabetic subjects with nonalcoholic fatty liver disease. Metab Syndr Relat Disord. 2015;13(7):319-25. doi: 10.1089/met.2015.0018.
31. Keating SE, Parker HM, Hickman IJ, Gomersall SR, Wallen MP, Coombes JS, et al. NAFLD in clinical practice: Can simple blood and anthropometric markers be used to detect change in liver fat measured by 1H-MRS? Liver Int. 2017;37(12):1907-15. doi: 10.1111/liv.13488.
32. Li L, You W, Ren W. The ZJU index is a powerful index for identifying NAFLD in the general Chinese population. Acta Diabetol. 2017;54(10):905-11. doi: 10.1007/s00592-017-1024-8.
33. Xu C, Ma Z, Wang Y, Liu X, Tao L, Zheng D, et al. Visceral adiposity index as a predictor of NAFLD: A prospective study with 4-year follow-up. Liver Int. 2018;38(12):2294-300. doi: 10.1111/liv.13941.
34. Fu CP, Ali H, Rachakonda VP, Oczypok EA, DeLany JP, Kershaw EE. The ZJU index is a powerful surrogate marker for NAFLD in severely obese North American women. PLoS One. 2019;14(11). doi: 10.1371/journal.pone.0224942.
35. Villanueva-Ortega E, Garcés-Hernández MJ, Herrera-Rosas A, López-Alvarenga JC, Laresgoiti-Servitje E, Escobedo G, et al. Gender-specific differences in clinical and metabolic variables associated with NAFLD in a Mexican pediatric population. Ann Hepatol. 2019;18(5):693-700. doi: 10.1016/j.aohep.2019.04.012.
36. Cen C, Wang W, Yu S, Tang X, Liu J, Liu Y, et al. Development and validation of a clinical and laboratory-based nomogram to predict nonalcoholic fatty liver disease. Hepatology International. 2020;14(5):808-16. doi: 10.1007/s12072-020-10065-7.
37. Okamura T, Hashimoto Y, Hamaguchi M, Obora A, Kojima T, Fukui M. The visceral adiposity index is a predictor of incident nonalcoholic fatty liver disease: A population-based longitudinal study. Clinics and research in hepatology and gastroenterology. 2020;44(3):375-83. doi: 10.1016/j.clinre.2019.04.002.
38. Zaki M, Amin D, Mohamed R. Body composition, phenotype and central obesity indices in Egyptian women with non-Alcoholic fatty liver disease. Journal of Complementary and Integrative Medicine. 2020;18(2):385-90. doi: 10.1515/jcim-2020-0073.
39. Li M, Shu W, Zunong J, Amaerjiang N, Xiao H, Li D, et al. Predictors of non-alcoholic fatty liver disease in children. Pediatr Res. 2021. doi: 10.1038/s41390-021-01754-6.

40. Lin IT, Lee MY, Wang CW, Wu DW, Chen SC. Gender differences in the relationships among metabolic syndrome and various obesity-related indices with nonalcoholic fatty liver disease in a taiwanese population. Int J Environ Res Public Health. 2021;18(3):1-13. doi: 10.3390/ijerph18030857.
41. Sheng G, Lu S, Xie Q, Peng N, Kuang M, Zou Y. The usefulness of obesity and lipid-related indices to predict the presence of Non-alcoholic fatty liver disease. Lipids Health Dis. 2021;20(1). doi: 10.1186/s12944-021-01561-2.

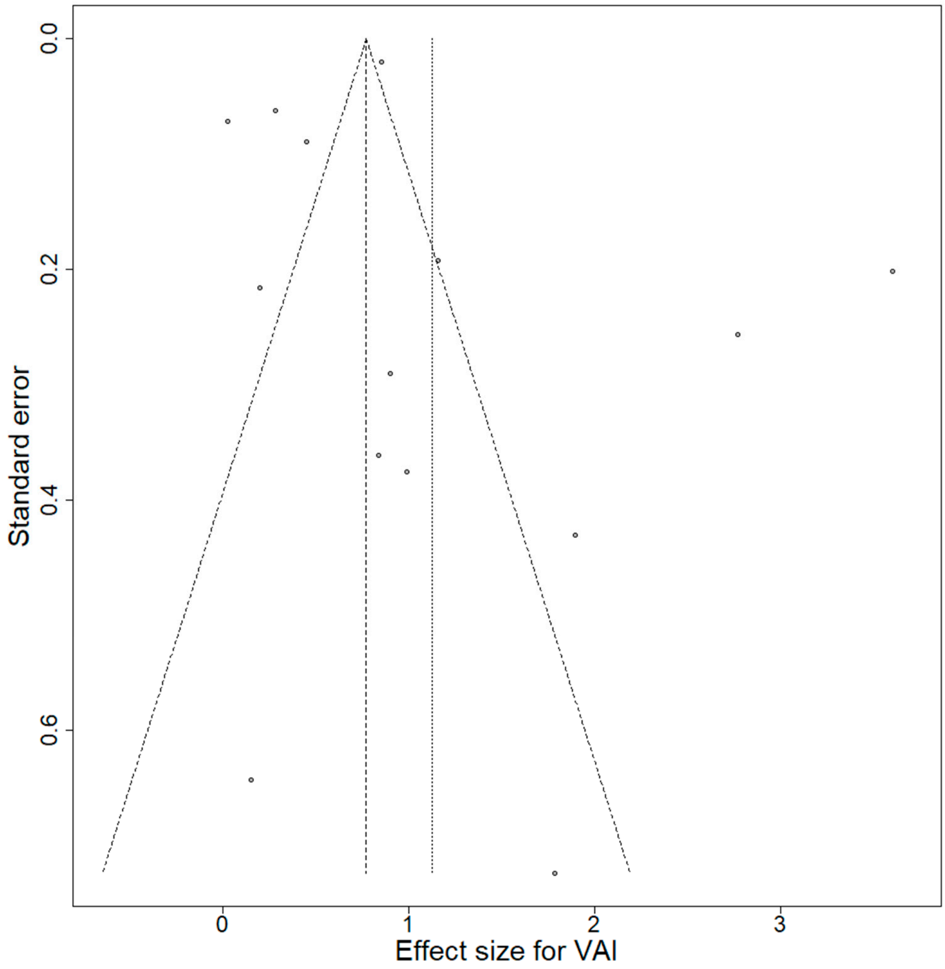

Supplementary Figure S1. Funnel plot for VAI, comparing adult and pediatric NAFLD patients with controls, with publication bias test (p-value=0.696).

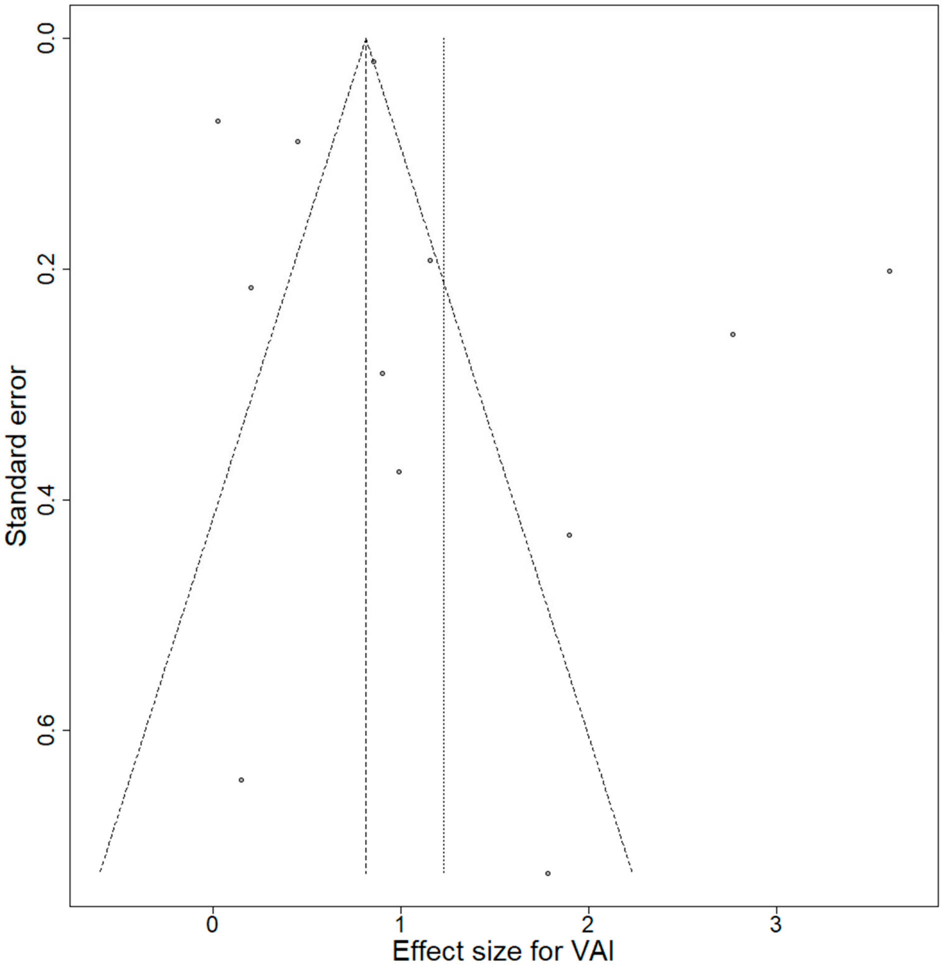

Supplementary Figure S2. Funnel plot for VAI, comparing adult NAFLD patients with controls, with publication bias test (p-value=0. 565).

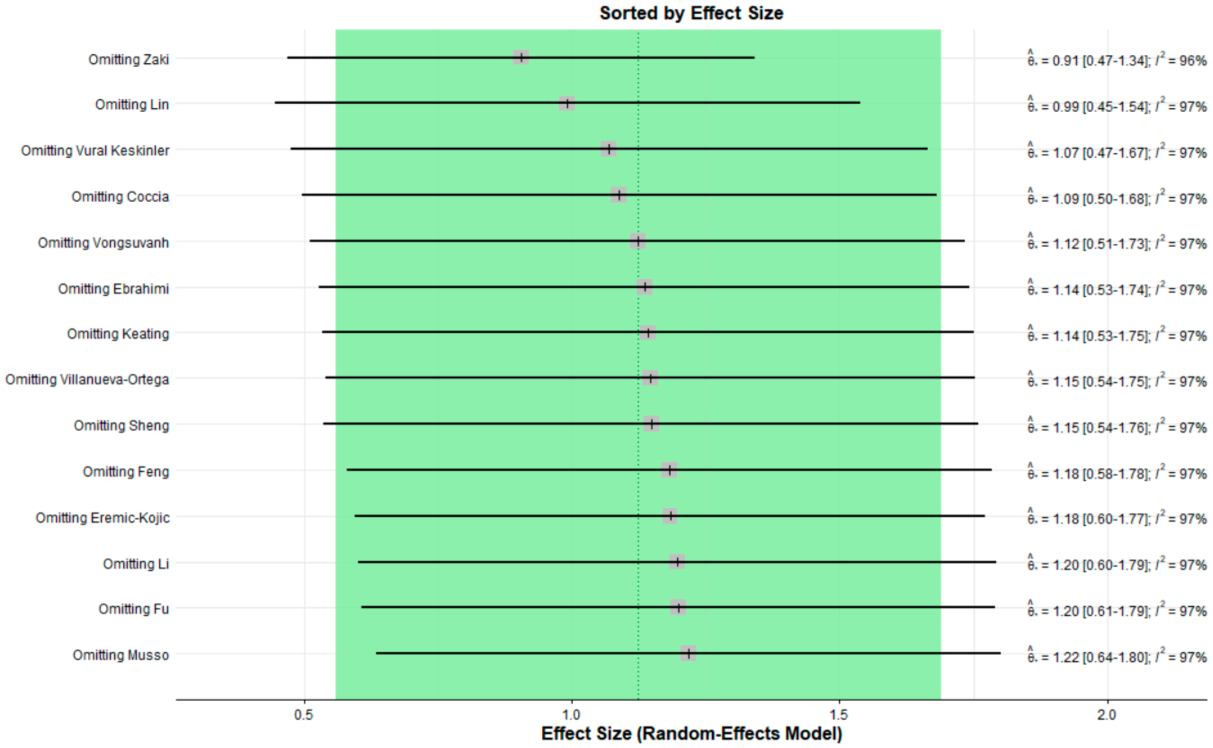

Supplementary Figure S3. Leave one out analysis for studies on adults and pediatrics showing the mean difference and I^2 along with 95% confidence intervals

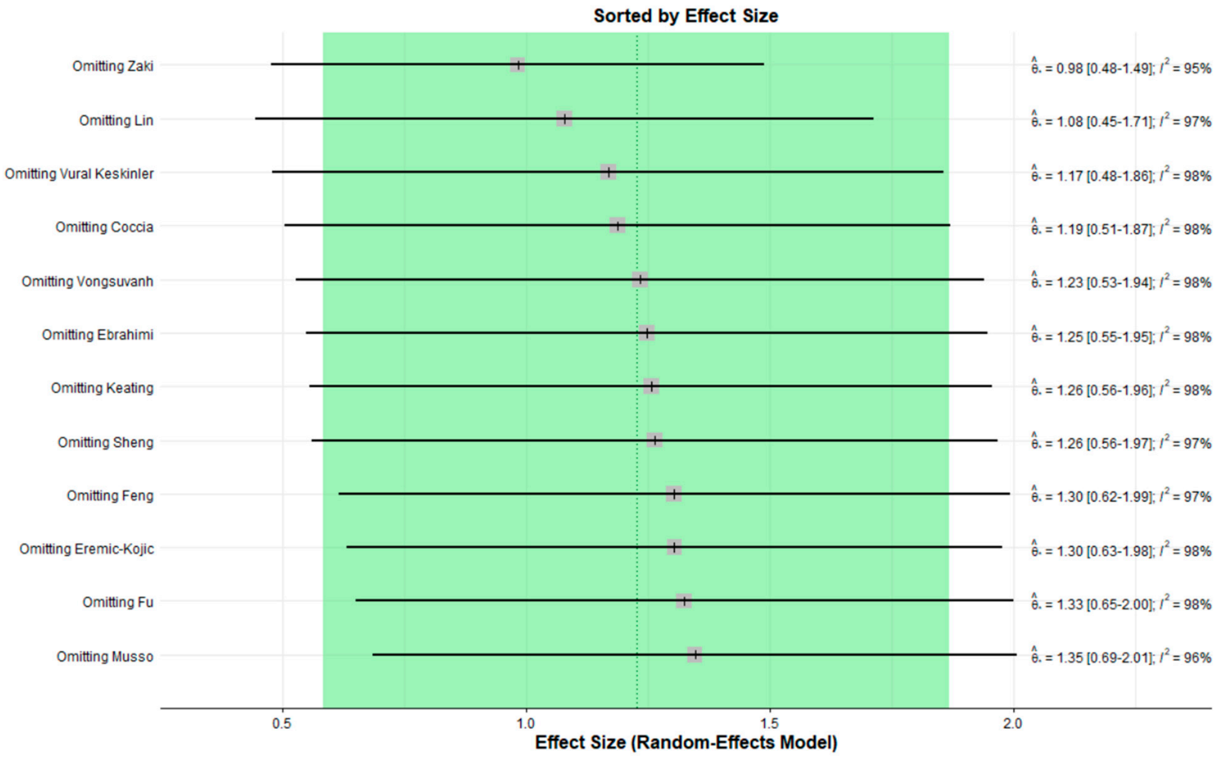

Supplementary Figure S4. Leave one out analysis for studies on adults showing the mean difference and I^2 along with 95% confidence intervals

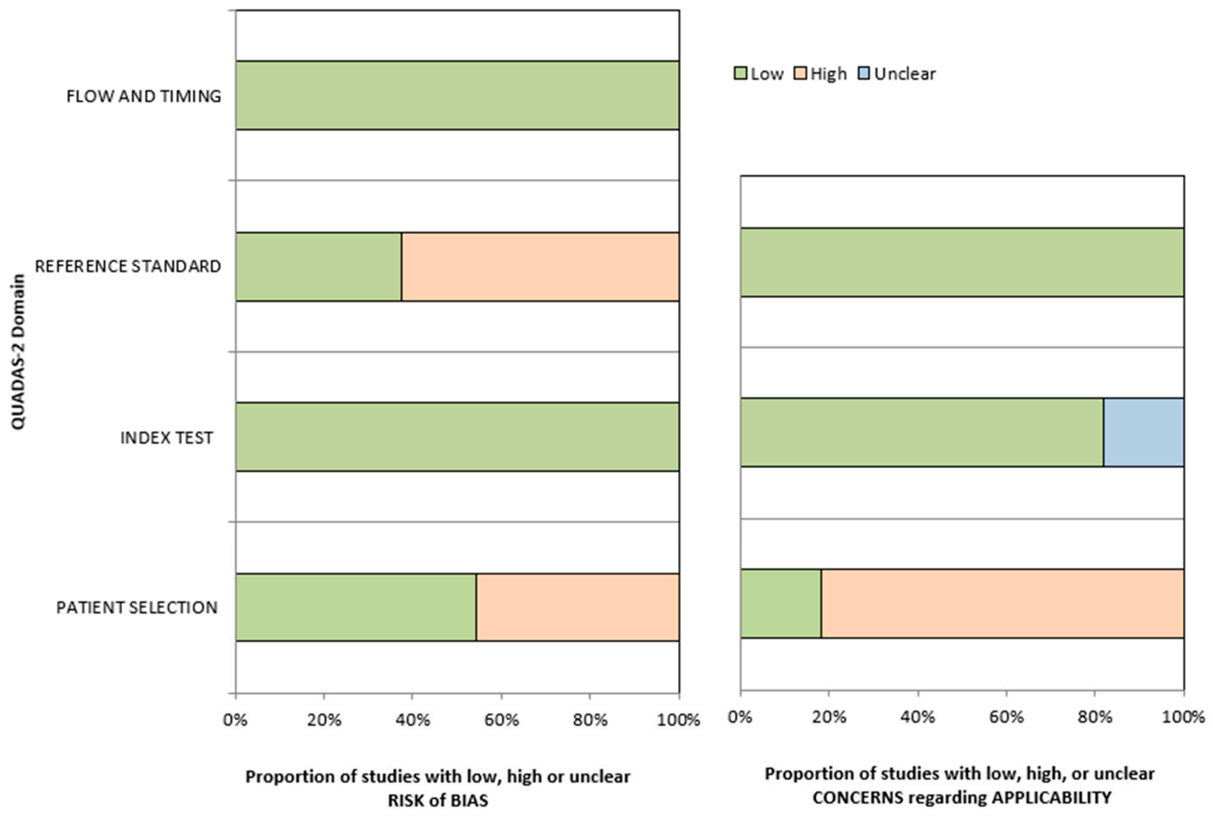

Supplementary Figure S5. Summary of the QUADAS 2 quality assessment according to the risk of bias and applicability of included studies
